# Unraveling dynamic immunological landscapes in intracerebral hemorrhage: insights from single‐cell and spatial transcriptomic profiling

**DOI:** 10.1002/mco2.635

**Published:** 2024-07-10

**Authors:** Lingui Gu, Hualin Chen, Mingjiang Sun, Yihao Chen, Qinglei Shi, Jianbo Chang, Junji Wei, Wenbin Ma, Xinjie Bao, Renzhi Wang

**Affiliations:** ^1^ Department of Neurosurgery Peking Union Medical College Hospital, Chinese Academy of Medical Sciences and Peking Union Medical College Beijing China; ^2^ Beijing Neurosurgical Institute, Beijing Tiantan Hospital, Capital Medical University Beijing China; ^3^ Research Institute of Big Data, Chinese University of Hong Kong (Shenzhen) School of Medicine Shenzhen China; ^4^ State Key Laboratory of Common Mechanism Research for Major Diseases Beijing China; ^5^ School of Medicine The Chinese University of Hong Kong Shenzhen Guangdong China

**Keywords:** intracerebral hemorrhage, single‐cell sequencing, spatial transcriptome, immune microenvironment, myeloid and lymphocytes

## Abstract

Intracerebral hemorrhage (ICH) poses a formidable challenge in stroke management, with limited therapeutic options, particularly in the realm of immune‐targeted interventions. Clinical trials targeting immune responses post‐ICH have encountered setbacks, potentially attributable to the substantial cellular heterogeneity and intricate intercellular networks within the brain. Here, we present a pioneering investigation utilizing single‐cell RNA sequencing and spatial transcriptome profiling at hyperacute (1 h), acute (24 h), and subacute (7 days) intervals post‐ICH, aimed at unraveling the dynamic immunological landscape and spatial distributions within the cerebral tissue. Our comprehensive analysis revealed distinct cell differentiation patterns among myeloid and lymphocyte populations, along with delineated spatial distributions across various brain regions. Notably, we identified a subset of lymphocytes characterized by the expression of Spp1 and Lyz2, termed macrophage‐associated lymphocytes, which exhibited close interactions with myeloid cells. Specifically, we observed prominent interactions between Lgmn+Macro‐T cells and microglia through the spp1–cd44 pathway during the acute phase post‐ICH in the choroid plexus. These findings represent a significant advancement in our understanding of immune cell dynamics at single‐cell resolution across distinct post‐ICH time points, thereby laying the groundwork for exploring critical temporal windows and informing the development of targeted therapeutic strategies.

## Graphical abstract

Using single‐cell transcriptomics, we explored post‐ICH cellular changes and intercellular dynamics. We identified distinct myeloid and lymphocyte subclasses. Early after ICH (≤24 h), myeloid cells dominated, while a lymphocyte subset showed heightened macrophage‐related markers. These findings highlight myeloid‐lymphocyte interactions, crucial in ICH pathogenesis. Further studies are warranted to explore subacute phase cellular engagements.

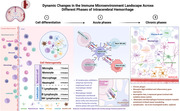

## 1 INTRODUCTION

Intracerebral hemorrhage (ICH) represents a multifaceted condition influenced by a myriad of genetic and environmental factors, thereby posing significant challenges for therapeutic intervention strategies. One key aspect crucial for addressing these challenges lies in comprehensively understanding the intricate interactions among immune cells throughout different stages of ICH progression.^1^ Advancements in stroke pathophysiology highlight the critical role of immune cells in modulating the cerebral immunological microenvironment.^2^ Given the brain's intricate cellular composition, comprising a diverse array of neuron and immune cell populations,^3^ elucidating the dynamic immune responses across various stages of stroke becomes paramount.

Single‐cell RNA sequencing (scRNA‐seq) is an essential high‐throughput approach, offering unprecedented insights into cellular diversity, lineage trajectories, intercellular interactions, and immune landscapes within the brain. Additionally, spatial transcriptomics (ST) and proteomics provide complementary tools for deciphering the spatial organization of cellular constituents and their molecular profiles, further enriching our understanding of complex biological processes. The emerging understanding of cellular diversity within the brain challenges conventional categorizations, such as the simplistic MG1‐MG2 classification of microglia (MG) activation,^4^ highlighting the nuanced roles played by immune cell populations in disease contexts.

Despite progress in single‐cell sequencing, the literature is sparse regarding the molecular and spatial dynamics post‐ICH at single‐cell resolution across various time points. Our study aims to fill this knowledge gap by pioneering the integration of single‐cell sequencing and ST to elucidate immune cell dynamics, interactions, and spatial distributions throughout stroke progression. By elucidating the immunological landscape at single‐cell resolution, our research aims to provide a significant resource that lays the groundwork for further deciphering the molecular and cellular underpinnings of stroke pathology.

## 2 RESULTS

### 2.1 Single‐cell and spatial transcriptome profiling of immune cells from brain tissue of rat at different stages after ICH

**FIGURE 1:**
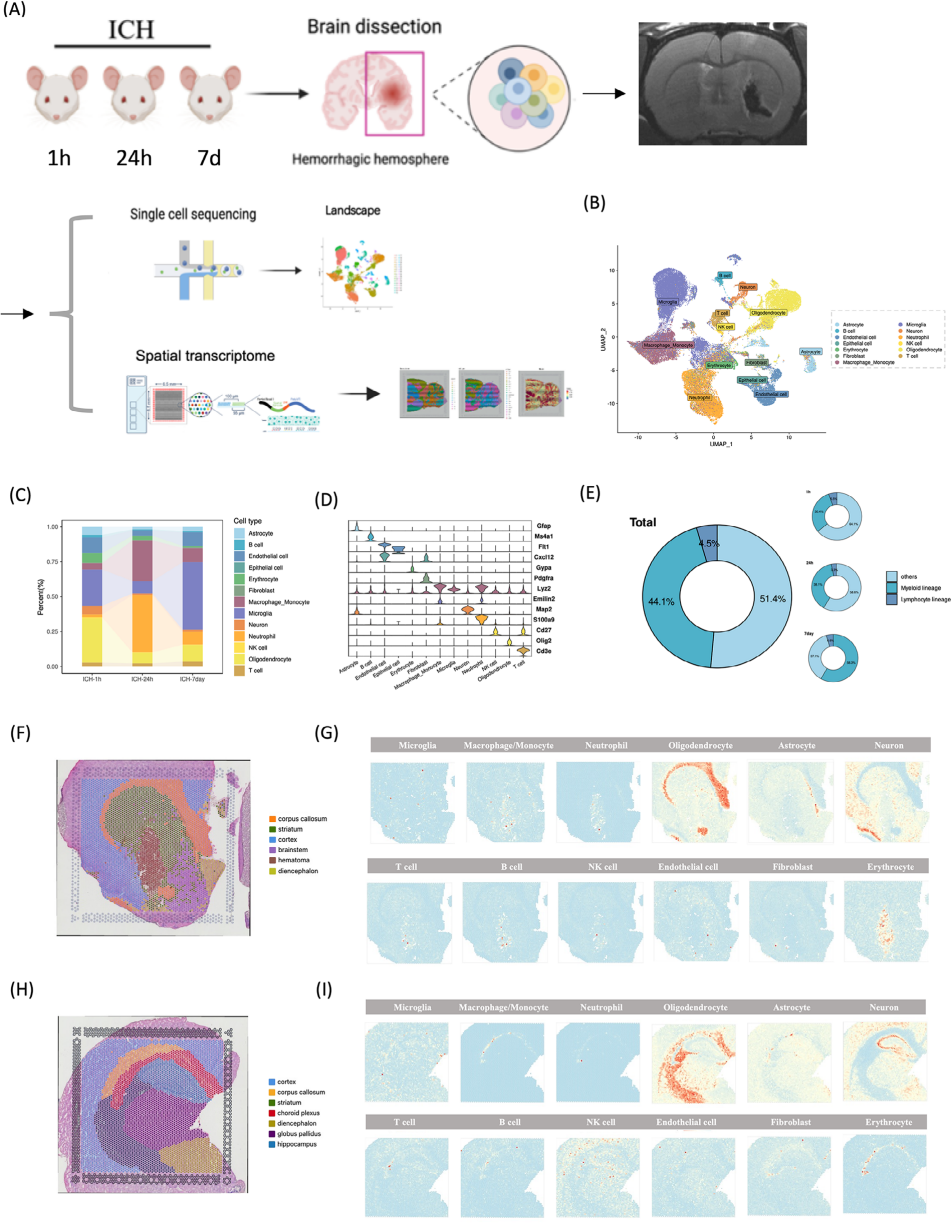
Single‐cell transcriptomic profiling of mouse brains after hemorrhagic stroke. (A) Experimental design for single‐cell RNA transcriptomic profiling of mouse brains after hemorrhagic stroke. Autologous blood stereotaxic injection surgical operations were performed to establish mouse models of ICH. Hemorrhagic hemispheres were collected at 1 h, 24 h, and 7 days poststroke. (B) UMAP visualization of a total of 63,211 cells colored by cell‐type annotation, clustered into 13 cell types based on core markers. (C) Bar plots showing the proportions of the 13 cell‐type subsets at each time point. (D) Violin plots showing average scaled RNA expression of that core markers in the cell type identified in (B). (E) Pie plots illustrating the proportions of myeloid and lymphocyte lineages at each time point. (F and H) Annotated cryosection on the ST slide. (G and I) All ST spots with cell types proportion inferred by CARD.

We performed scRNA‐seq on nine brain hemispheres from young adult rats (8–10 weeks old) at distinct post‐ICH stages: 1 h, 24 h, and 7 days post‐ICH (*n* = 3 per group), induced via autologous blood stereotaxic injection, to delineate the spatiotemporal transcriptomic alterations in individual cells (Figure 1A). MRI scan was performed on cerebral hemispheres (Figure 1A). We then annotated the cells using SingleR and CellMarker and identified 13 cell types based on their core markers (Figure 1B,D), including MG, macrophages and monocytes, endothelial cells, epithelial cells, erythrocyte, oligodendrocytes, neutrophils, T cells, astrocytes, nature killer cells (NKs), B cells (B), fibroblast cells, and neurons. Subdivide the results according to the samples and label each subgroup according to the samples. We examined the proportion of cells at different time periods and discovered that the proportion of cells at different time points following ICH differed (Figure 1C). Following that, we undertook the endeavor of classifying immune cells into myeloid (MG, macrophage/monocyte, and neutrophiles) and lymphoid cells (T, B, NK cells). Our observations revealed a noteworthy disparity in the distribution of myeloid and lymphoid cells across the hyperacute, acute, and subacute repair stages following ICH. Myeloid cells, which are considerably more abundant than lymphoid cells after ICH, predominate (Figure 1E). We applied hierarchical clustering to elucidate the spatial organization of cells at 1 and 24 h post‐ICH, revealing distinct clusters that correspond to known anatomical regions: cortex, striatum, choroid plexus, diencephalon, globus pallidus, hematoma, and hippocampus (Figure 1F,H). From the types of proportion inferred by Cellular Abundance and RNA Detection (CARD), we observed the distribution of various cells in 1 and 24 h post‐ICH (Figure 1G,I) As expected in previous studies, neurons were present in both the cortex and striatum not just at the 1 h but also at the 24 h post‐ICH. Immune cell populations, such as MG, macrophages/monocytes, and neutrophils, exhibited increased abundance in the striatum and choroid plexus. Lymphoid cells, including T, B, and NK cells, were seen to be present in the hematoma region within 1 h after ICH. However, their predominant accumulation was noted in the choroid plexus at 24 h after ICH.

### 2.2 Two MG subpopulations with apparently opposite features emerge at different stages after ICH

**FIGURE 2:**
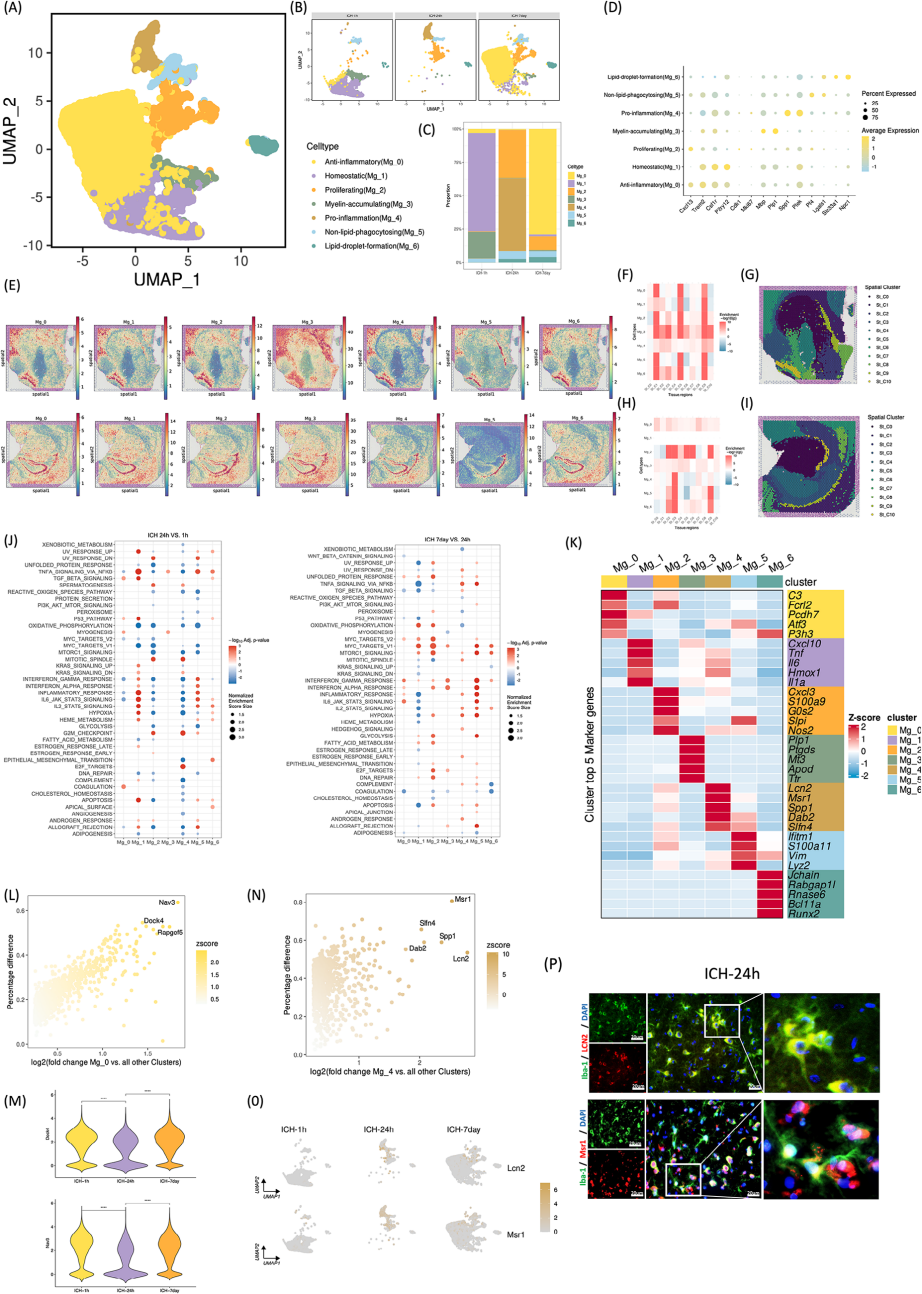
Distinct subclusters of the myeloid lineage in microglia cluster. (A) UMAP visualization of a total of 18,938 microglia, annotated and colored based on clustering. (B) UMAP visualization of the single‐cell transcriptomic profiles of seven microglia subclusters in (A) at each time point. (C) Bar plots showing the proportions of the seven microglia subclusters at each time point. (D) Bubble Chart showing the classical markers of microglia subclusters. (E) Estimated cell abundances (color intensity) of seven microglia subclusters across regions of rat brains at ICH‐1 h (up) and ICH‐24 h (bottom). (F–I) The MIA map of microglia subtypes unraveled by scRNA‐seq and ST‐defined histological regions. Red indicates enrichment, whereas blue indicates depletion (up: 1 h post‐ICH, down: 24 h post‐ICH). (J) Top enriched GO‐BP terms of marker genes of DEGs were illustrated at 24 h (vs. 1 h, left) and 7day (vs. 24 h, right). (K) Heatmap displaying the average expression levels of the top 5 marker genes in each microglia subcluster. (L–O) Scatter plots showing the upregulated DEGs of Mg_0 (L) or Mg_4 (N) compared with all other microglia subclusters. The *y*‐axis represents the percentage difference (Δ percent of cells), and the *x*‐axis represents the log‐fold change (measured by Wilcoxon rank‐sum test). Each dot corresponds to a DEG, color‐coded based on the combined *Z*‐score of the percentage difference and log‐fold change. Dots with high *Z*‐scores are labeled. (M) Violin plots illustrating the expression level differences of Nav3 (up) and Dock4 (down) between time points. (O) UMAPs as in (B) but colored by expressions of Lcn2 (up) and Msr1 (bottom). (1 h vs. 24 h and 7day vs. 24 h, by Wilcoxon rank‐sum tests. ****p < 0.0001. (P) Double immunofluorescence staining showed LCN2 and MSR1 expressed on microglia at 24 h after ICH, respectively. Scale bar = 20 µm. *n* = 3 for each group.

MG heterogeneity following hemorrhagic stroke was comprehensively investigated through clustering analysis. Initially, unsupervised clustering of MG from total cells delineated seven distinct subclusters: anti‐inflammatory (Mg_0), homeostatic (Mg_1), proliferating (Mg_2), myelin‐accumulating (Mg_3), proinflammation (Mg_4), nonlipid‐phagocytosing (Mg_5), and lipid‐droplet‐formation (Mg_6) (Figure 2A,D,K). Visualization using Uniform Manifold Approximation and Projection (UMAP) depicted temporal changes in MG subpopulations (Figure 2B) and facilitated quantification of their proportionate shifts over time (Figure 2C). ST analysis revealed predominant microglial localization in the choroid plexus at 1 and 24 h post‐ICH (Figure 2E). Multimodal intersection analysis (MIA) integrating scRNA‐seq and spRNA‐seq elucidated the distribution of MG subtypes at 1 and 24 h post‐ICH (Figure 2F‐I). The Mg_4 subgroup predominated at 24 h post‐ICH, exhibiting notable upregulation of proinflammatory markers Lcn2 and Msr1 (Figure 2K,N,O). Conversely, the Mg_0 subgroup was enriched during the subacute phase (7 days) poststroke, displaying elevated expression of anti‐inflammatory markers C3, Fcrl2, Pcdh7, Atf3, and P3h3 (Figure 2L,M). Immunofluorescence validation confirmed the expression patterns of Lcn2 and Msr1 on MG in the hemorrhagic stroke rat brain 24 h after ICH (Figure 2P). These findings underscore the dynamic nature of microglial responses post‐ICH and highlight distinct phenotypic shifts associated with different stages of stroke progression.

### 2.3 Detecting neutrophil subset differentiation after ICH

**FIGURE 3:**
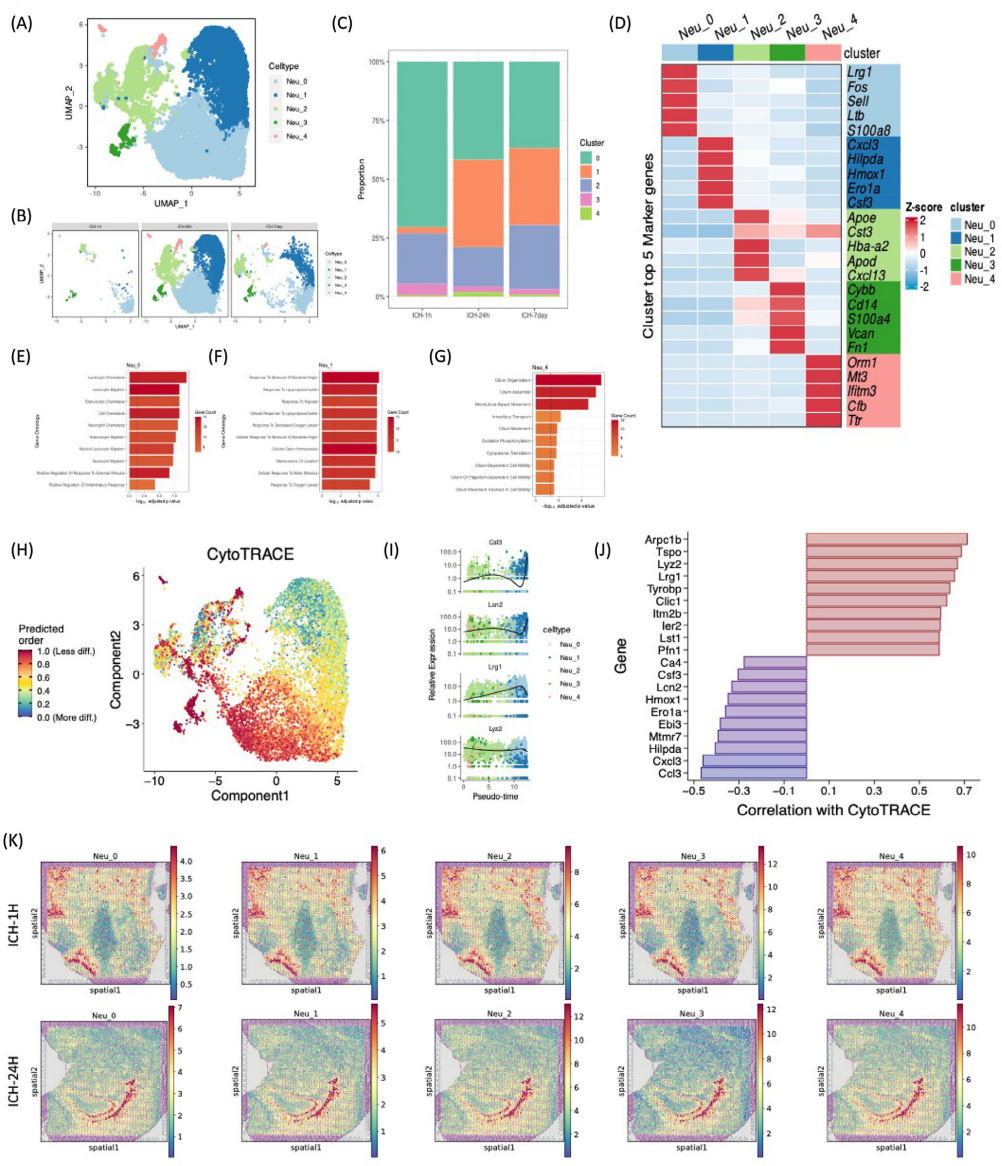
Neutrophils transcriptional changes through hemorrhagic stroke. (A) UMAP visualization of a total of 10,538 neutrophils, annotated and colored based on clustering. (B) UMAP visualization of the single‐cell transcriptomic profiles of five neutrophils subclusters in (A) at each time point. (C) Bar plots showing the proportions of the five neutrophils subclusters at each time point. (D) Heatmap displaying the average expression levels of the top 5 marker genes in each neutrophil subcluster. (E–G) Top enriched GO‐BP terms of marker genes of Neu_0 (E), Neu_1 (F), and Neu_4 (G). (H) UMAP as in (A) but colored by the cell differentiation potentials precited by CytoTRACE. Cell differentiation potential from low (1.0, Less diff.) to high (0, High diff.) are indicated by a color gradient from red to blue. (I) Top genes correlated with less differentiated and more differentiated neutrophils predicted by CytoTRACE. (J) The relative expression levels of indicated genes along with the pseudotime inferred by Monocle. (K) Estimated cell abundances (color intensity) of five neutrophils subclusters across regions of rat brains at ICH‐1 h (up) and ICH‐24 h (bottom).

The peak percentage of neutrophils occurred 24 h post‐ICH (Figure 1C). Our investigation identified five distinct neutrophil clusters, labeled Neu_0 to Neu_4. Notably, the Neu_0 subgroup predominated among stroke neutrophils at 1 h, 24 h, and 7 days, exhibiting heightened expression of chemotactic genes such as Lrg1, Fos, Sell, Lfb, and S100a8 (Figure 3A–D). Conversely, the Neu_1 subgroup demonstrated significant proliferation at 24 h and 7 days post‐ICH, contrasting with the 1‐h time point. This expansion correlated with increased expression of cytokine‐responsive genes, specifically Cxcl3, Hilpda, Hmox1, Ero1a, and Csf3 (Figure 3C,D,F). Intriguingly, the transient emergence of the Neu_4 subgroup at 24 h post‐ICH, followed by its decline at 7 days, was observed (Figure 3C). The Neu_4 subgroup exhibited a unique transcriptomic signature, marked by upregulation of genes involved in oxidative stress mitigation, including ORM1, MT3, IFITM3, CFB, and TTR, indicative of a neuroprotective function following ICH. Further analysis revealed differentiation trajectories between developmental stages, with the Neu_0 subgroup exhibiting the highest CytoTRACE scores among all neutrophil subpopulations, indicative of limited differentiation and suggestive of an “immature Neu_0” phenotype (Figure 3H). High‐differentiation genes included Arpc1b, Tspo, Lyz2, Lrg1, Tyrobp, Clic1, Itm2b, Ler2, Lst1, and Pfn1 (Figure 3I,J). Additionally, examination of neutrophil distribution demonstrated similar enrichments in the choroid plexus at 1 h post‐ICH, with heightened localization in the choroid plexus at 24 h (Figure 3K). These findings underscore the dynamic nature of neutrophil responses following ICH, revealing distinct functional states and spatial dynamics across different post‐ICH time points.

### 2.4 Heterogeneity and cell differentiation of macrophages/monocytes during different stages after ICH

**FIGURE 4:**
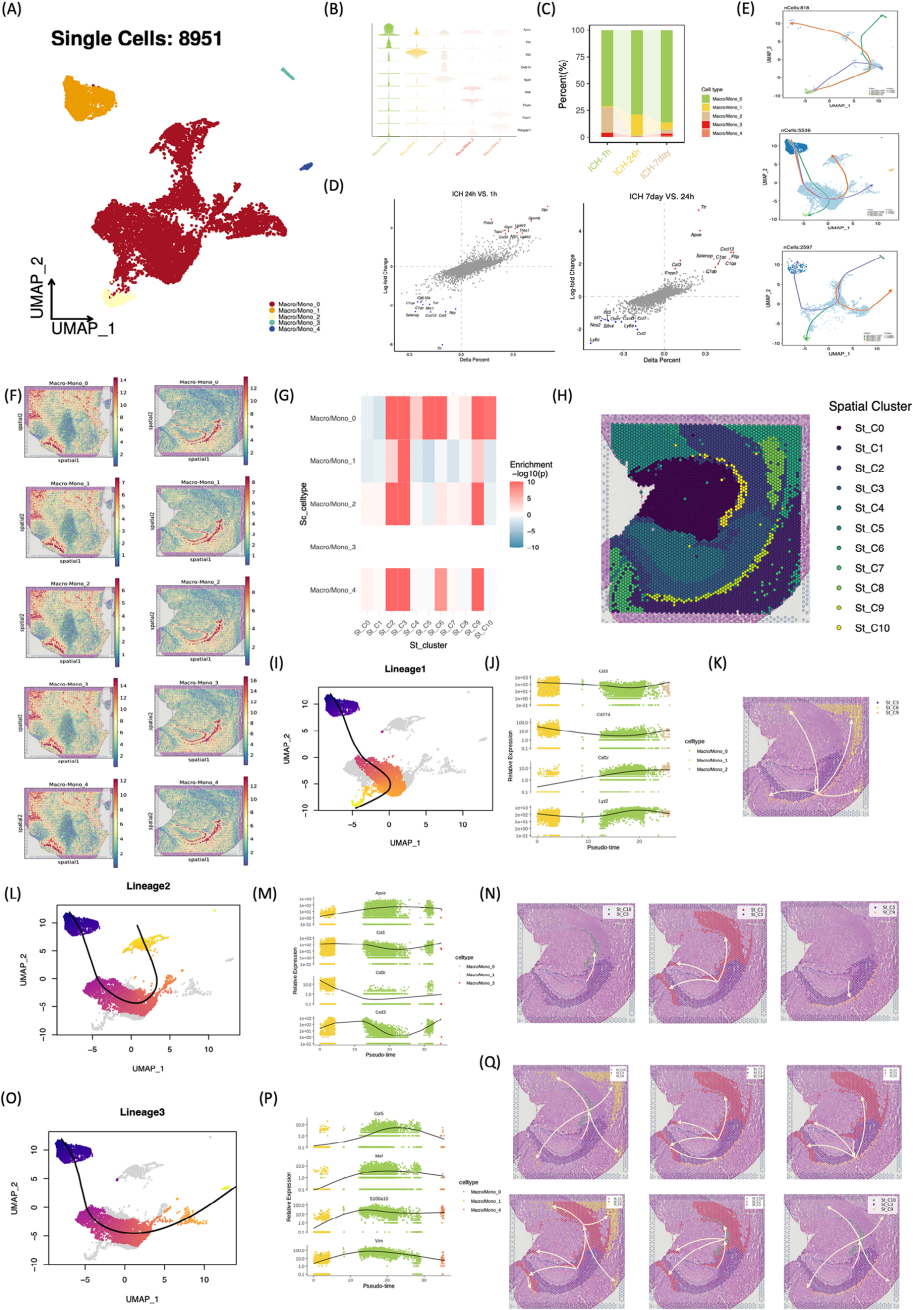
Heterogeneity and cell differentiation of macrophages/monocytes during different stages after ICH. (A) UMAP visualization of Macro/Mono annotated and colored based on clustering. (B) The expression levels of cell‐type specific markers across each subset. (C) Bar plots showing the proportions of each subset at each time point. (D) DEGs at 24 h (vs. 1 h, left panel) and 7 day (vs. 24 h, right panel). The *x*‐axis represents the percentage difference (Δ percent of cells), and the *y*‐axis represents the log‐fold change (measured by Wilcoxon rank‐sum test). (E) Developmental trajectories at 1 h (upper panel), 24 h (middle panel), and 7d (bottom panel). (F) Estimated cell abundances (color intensity) of each subset across regions of rat brains at ICH‐24 h. (G) The spatial map of Macro/Mono subsets unraveled by scRNA‐seq and ST‐defined histological regions by MIA method. Red indicates enrichment, whereas blue indicates depletion. (H) The spatial subclusters identified by the Seurat analytic pipeline under the resolution of 0.6. (I–Q) Integration of single‐cell and spatial trajectory inferences. (I, L, and O) The inferred three trajectory (termed as Lineage 1, 2 and 3) of Macro/Mono subsets at 24 h, corresponding to Figure 4E (middle panel). (J, M, and P) The dynamic single‐cell expression profiles of certain biomarkers along with the pseudotime. (K, N, and Q) Reconstruction of the spatial trajectory based on the spatial information and gene expression profile.

The intricate interplay among macrophages/monocytes (Macro/Mono), pivotal in sensing and responding to inflammation and tissue damage, prompted an in‐depth investigation into their potential interactions. Five distinct subsets of Macro/Mono were delineated (Figure 4A,B). Notably, Macro/Mono_0 emerged as the predominant subset at 24 h post‐ICH, while Macro/Mono_1 exhibited transient presence at 24 h, diminishing at 1 h and 7 days post‐ICH (Figure 4C), warranting further attention. Differential expression analysis of macrophage/monocyte genes revealed distinct profiles across acute (24 h) and subacute (7 days) phases post‐ICH. Specifically, macrophages/monocytes in acute stages displayed upregulation of Cxcl3, Slpi, Gpnmb, Spp1, Fcgr2b, CD63, Lgals3, and Prdx5. Cxcl3 is a proinflammatory mediator, highly expressed in inflammatory macrophage.^5^ Spp1, a multifunctional and highly phosphorylated glycoprotein, is highly expressed in macrophage and associated with inflammation.^6^ Subacute phases exhibited unique expression patterns, including upregulation of Apoe, Cxcl13, Pltp, C1qc, and Ttr (Figure 4D). Transthyretin (TTR) is recognized as a quintessential neuroprotective agent and a suppressor of oxidative stress.^7^ Spatial analysis revealed a concentration of Macro/Mono in the choroid plexus at 24 h post‐ICH (Figure 4F–H). To delineate the differentiation trajectory of Macro/Mono post‐ICH, Slingshot analysis was employed to re‐order the subsets based on the dynamic transcriptional profiles (Figure 4E). At 24 h post‐ICH, three distinct differentiation lineages were inferred, with Macro/Mono_0 initiating the differentiation process, transitioning to Macro/Mono_1, and subsequently giving rise to Macro/Mono_2, Macro/Mono_3, and Macro/Mono_4, each exhibiting distinct gene expression profiles (Figure 4I,J,L,M,O,P). Integration of scRNA‐seq and spRNA‐seq^8^ through MIA facilitated spatial trajectory analysis using the stLearn^9^ toolkit, revealing trajectory and pseudo‐time analysis applied to spatial‐seq data (Figure 4K,N,Q). Macrophages distributed in different regions of the brain perform different functions.^10^, ^11^ These spatial trajectories inferred that Macro/Mono that are relatively low in development at the beginning of the process are almost always clustered in the choroid plexus regions, while the more mature cells all migrate distally to the striatum and cerebral cortex.

### 2.5 Single‐cell transcriptomics of subclustered T lymphocytes

**FIGURE 5:**
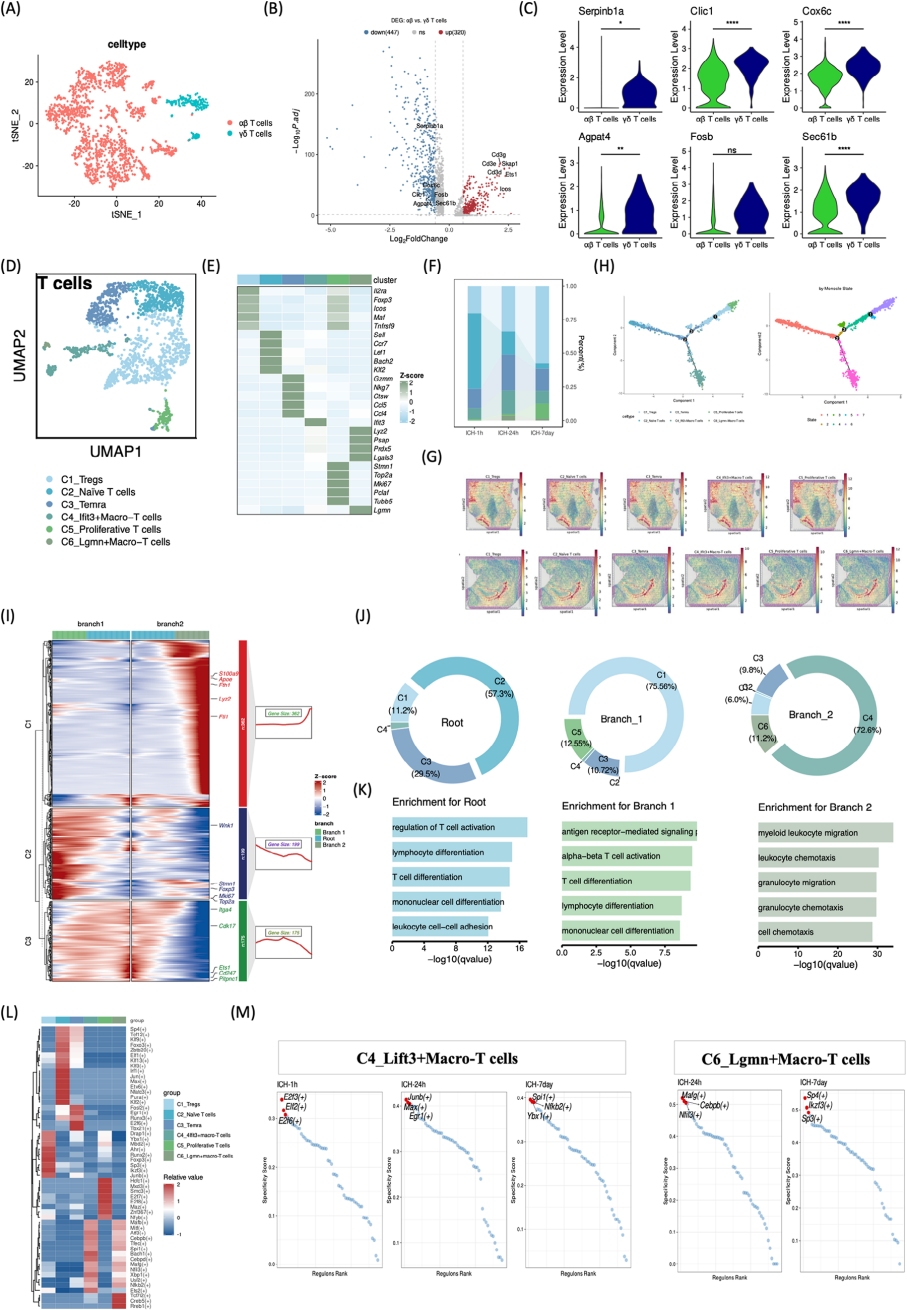
Identification of two major subclusters of T cells in rat brain post hemorrhagic stroke. (A) UMAP visualization of a total of 1836 T cells in Figure 1B, clustered into αβ and γδ T cells. (B) Volcano plot showing the DEGs between αβ and γδ T cells. Cell‐type specific markers retrieved from literature were annotated in the DEGs. (C) Violin plots illustrating the expression level differences of cell‐type specific markers in (B) between αβ and γδ T cells by Wilcoxon rank‐sum tests. *p <0.05, **p < 0.01, ****p < 0.0001, ns not significant. (D) UMAP visualization of a total of 1613 αβT cells, annotated and colored based on clustering. (E) Heatmap displaying the average expression levels of the top 5 marker genes in each T‐cell subcluster. (F) Bar plots showing the proportions of the six T‐cell subclusters at each time point. (G) Estimated cell abundances (color intensity) of αβT subclusters across regions of rat brains at ICH‐1 h (up) and ICH‐24 h (bottom). (H) Pseudotime trajectory trees showing the ordering of T‐cell clusters marked by Seurat (left) or Monocle (right). (I) BEAM analysis of branch point 2 revealed three clusters of branch‐dependent genes. Heatmap on the left demonstrating the transcriptional changes of these genes from root to branch 1/2. The gene counts and the selected five genes of each gene cluster were shown on the right. The gene expression was *Z*‐score transformed. (J) Pie plots showing the distribution of T‐cell clusters in each defined Monocle state (Root: State 1; Branch 1: States 2−6; Branch 2: State 7). C1–C6 represent the prefixes of T‐cell subclusters. (K) Bar plots illustrating the selected top GO‐BP terms related to the three clusters of branch‐dependent genes in (I). (L) Heatmap presenting the average regulon activities in each T‐cell subcluster, with colors ranging from blue to red indicating relative expression levels, from low to high. (M) Dot plots showing the specialty score of each regulon of C4_Ifit3+Macro T cells and C6_Lgmn+Macro T cells at each time point. The top three regulons with highest specialty score were annotated.

T lymphocytes were classified into αβ and γδ subsets based on T cell receptor markers.^12^ T cells were categorized as αβ (Skap1, Ets1, Cd3g, Lcos, Cd3e, Cd3d) and γδ (Serpinb1a, Clic1, Cox6c, Agpat4, Fosb, Sec61b) using canonical markers (Figure 5A–C). Differentially expressed gene (DEG) demonstrated elevated levels of CD3G, SKAP1, ETS1, LCOR, CD3E, and CD3D in αβ T cells relative to γδ T cells (Figure 5B). Sub clustering of αβ T cells unraveled six distinct marker gene‐defined subclusters, namely C1_Tregs, C2_Navie T cells, C3_TEMRA, C4_Lift3+Macro‐T cells, C5_Proliferative T cells, and C6_Lgmn+Macro‐T cells (Figure 5D,E). Remarkably, the C4 and C6 subsets, designated as macrophage‐related lymphocytes, exhibited high expression of macrophage‐related markers, Lyz2 and Spp1. Immunofluorescence was performed to validate the coexpression of CD3 and Lyz2, CD3 and Spp1 in rat brain tissue after ICH (Figure S3B). Temporal analysis revealed an increase in Tregs and proliferative T cells from the super‐acute (1 h) to subacute (d7) phases post‐ICH, while acute TEMRA, Lift3+Macro‐T, and Lgmn+T cell proportions consistently surpassed those of hyperacute and subacute recovery phases (Figure 5F). ST illustrated T cell enrichment in the choroid plexus at 24 h post‐ICH (Figure 5G). Two T cell gene expression branch points were found in pseudotemporal trajectory analysis. Subclusters were projected onto the pseudotime plot, showing that C2_Navie T cells and C3_TEMRA were at the root, C1_Tregs and C5_Proliferative T cells were on branch 1, and C4_Lift3+Macro‐T cells and C6_Lgmn are on branch 2 (Figure 5H–J). The root state also expressed more Itga4, Cdk17, Ets1, Cd247, and Pitpnc1 genes, according to monocle analysis. In branch 1, Wnk1, Stmn1, Foxp3, Mki67, and Top2a were substantially expressed; in branch 2, S100a9, Apoe, Fth1, Lyz2, and Ftl1 were. Root state genes were linked to T cell activation, differentiation, mononuclear cell differentiation, and cell–cell adhesion by functional enrichment analysis. Antigen receptor‐mediated signaling, T cell activation, and differentiation were concentrated in branch 1 genes, whereas myeloid leukocyte movement, chemotaxis, and cell migration were in branch 2 genes (Figure 5J,K). There were also different transcription factor (TF) patterns in each T cell subtype. Drap1, Ybx1, Mbd2, Ahr, Runx2, Foxp3, and others were expressed more in C1_Tregs. Sp4, Tcf12, Klf9, Foxo3, Zbtb20, Elf1, and others were increased in C2_Navie T cells. Fosl2, Egr1, Runx3, E2f6, Tbx21, and others were greater in C3_TEMRA. E2f3, Elf2, E2f6, Junb, Max, Egr1, and others were expressed greater in C4_Lift3+Macro‐T cells. Hcfc1, Mxd3, Smc3, E2f7, E2f8, Maz, Znf367, Nfyb, and others were expressed greater in C5_Proliferative T cells. Tcf7l2, Creb5, and Rreb1 were greater in C6_Lgmn+Macro‐T cells (Figure 5L). Three major regulators of C4_Lift3+Macro‐T cells following ICH were E2f3, Elf2, E2f6 at super‐acute phases (1 h), Junb, Max, Egr1 at acute phases (24 h), and Spi1, Nfkb2, Ybx1 at subacute phases (d7). Mafg, Cebpb, Nfil3, SP4, Lkzf3, and Sp3 only revealed themselves at 24 h and d7, respectively, regulating 6_Lgmn+Macro‐T cells (Figure 5M).

### 2.6 Identification of macrophage‐associated B lymphocytes unveiled by single‐cell transcriptomics

**FIGURE 6:**
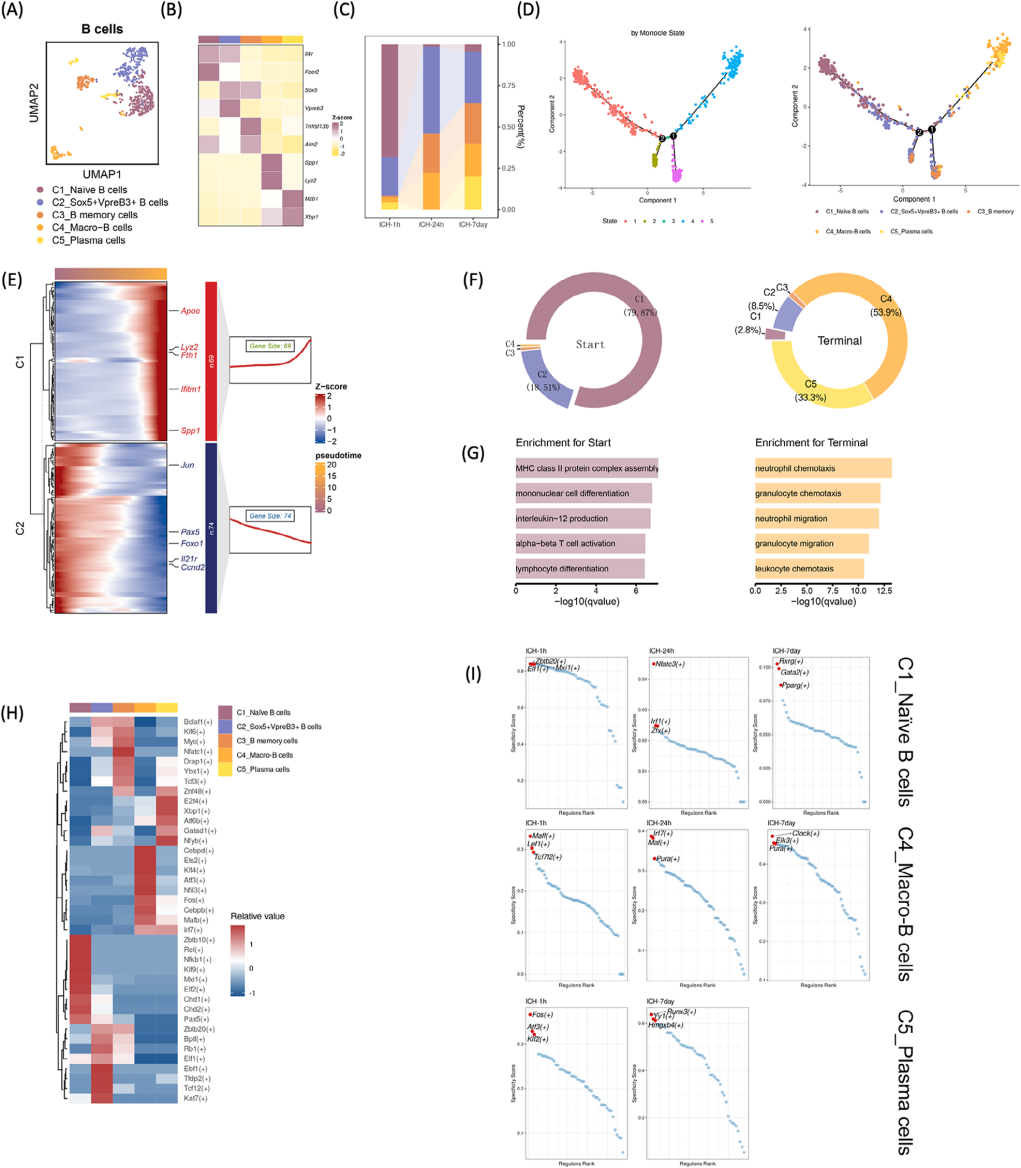
Trajectory of the B cells post hemorrhagic stroke. (A) UMAP visualization of a total of 685 B cells in Figure 1B, annotated and colored based on clustering. (B) Heatmap displaying the average expression levels of the top two marker genes in each B‐cell subcluster. (C) Bar plots showing the proportions of the five B‐cell subclusters at each time point. (D) Pseudotime trajectory trees showing the ordering of B‐cell clusters marked by Seurat (left) or Monocle (right). (E) Pseudotime analysis revealed two clusters of branch‐dependent genes. Heatmap on the left demonstrating the transcriptional changes of these genes from start to terminal. The gene counts and the selected five genes of each gene cluster were shown on the right. The gene expression was *Z*‐score transformed. (F) Pie plots showing the distribution of B‐cell clusters in each defined Monocle state (Start: State 1; Terminal: State 4). C1–C5 represent the prefixes of B‐cell subclusters. (G) Bar plots illustrating the selected top GO‐BP terms related to the two clusters of branch‐dependent genes in (E). (H) Heatmap presenting the average regulon activities in each B‐cell subcluster, with colors ranging from blue to red indicating relative expression levels, from low to high. (I) Dot plots showing the specialty score of each regulon of C1_ Naïve B cells, C4_ Macro B cells, and C5_Plasma cells at each time point. The top three regulons with highest specialty score were annotated.

We identified five B lymphocyte subclusters using higher expression markers: C1‐Naïve (IL‐4r, Fcer2), C2_Sox5+Vpreb3+ (Sox5, Vpreb3), C3_B memory (Tnfrsf13b, Aim2), C4_Macro‐B (Spp1, Lyz2), and C5_Plasma (Mzb1 Xbp1) (Figure 6A,B). During acute stages post‐ICH, C2_Sox5+Vpreb3+ B cells, C3_B memory cells, and C4_Macro‐B cells (Spp1, Lyz2) exhibited heightened proportions, while C5_Plasma cells were relatively scarce but gradually increased throughout the subacute phase post‐ICH (Figure 6C). The pseudotemporal order determined by diffusion pseudotime analysis aligned with the differentiation phases of these clusters. Monocle pseudotime plots positioned C1_Naïve B cells proximally to the root and C4_Macro‐B and C5_Plasma cells nearer to the terminal point (Figure 6D). Monocle analysis revealed two pseudotime‐dependent gene clusters progressing from the root to the terminal state. Genes such as Jun, Pax5, Foxo1, Il21r, and Ccnd2 were notably expressed in the root, whereas Apoe, Lyz2, Fth1, Ifitm1, and Spp1 were prominent in the terminal state (Figure 6E). In the root state, C1‐Naïve and C2_Sox5+Vpreb3+ B cells predominated, with gene enrichment related to MHC class II protein complex assembly, mononuclear cell differentiation, interleukin‐12 synthesis, alpha‐beta T cell activation, and lymphocyte differentiation. Conversely, C4_Macro‐B and C5_Plasma cells dominated the terminal state, with enrichment observed in neutrophil, granulocyte, neutrophil, and leukocyte chemotaxis genes (Figure 6F,G). Examination of TFs in each cell subtype revealed distinct regulatory profiles. Notably, C1‐Naïve B cells exhibited elevated levels of Zbtb10, Rel, Nfkb1, Klf9, Mxi1, Elf2, Chd1, Chd2, and Pax. Conversely, C4_Macro‐B cells demonstrated higher expression of Cebpd, Ets2, Klf4, Atf3, Nfil3, Fos, Cebpb, Mafb, and Irf7, while C5_Plasma cells displayed elevated levels of Znf48, E2f4, Xbp1, Atf6b, Gatad1, and Nfy. Following ICH, regulatory networks governing each B cell subtype varied across super‐acute, acute, and subacute phases, highlighting dynamic regulatory mechanisms orchestrating B cell responses post‐ICH (Figure 6H,I).

### 2.7 Identification of macrophage‐associated NK lymphocytes unveiled by single‐cell transcriptomics

**FIGURE 7:**
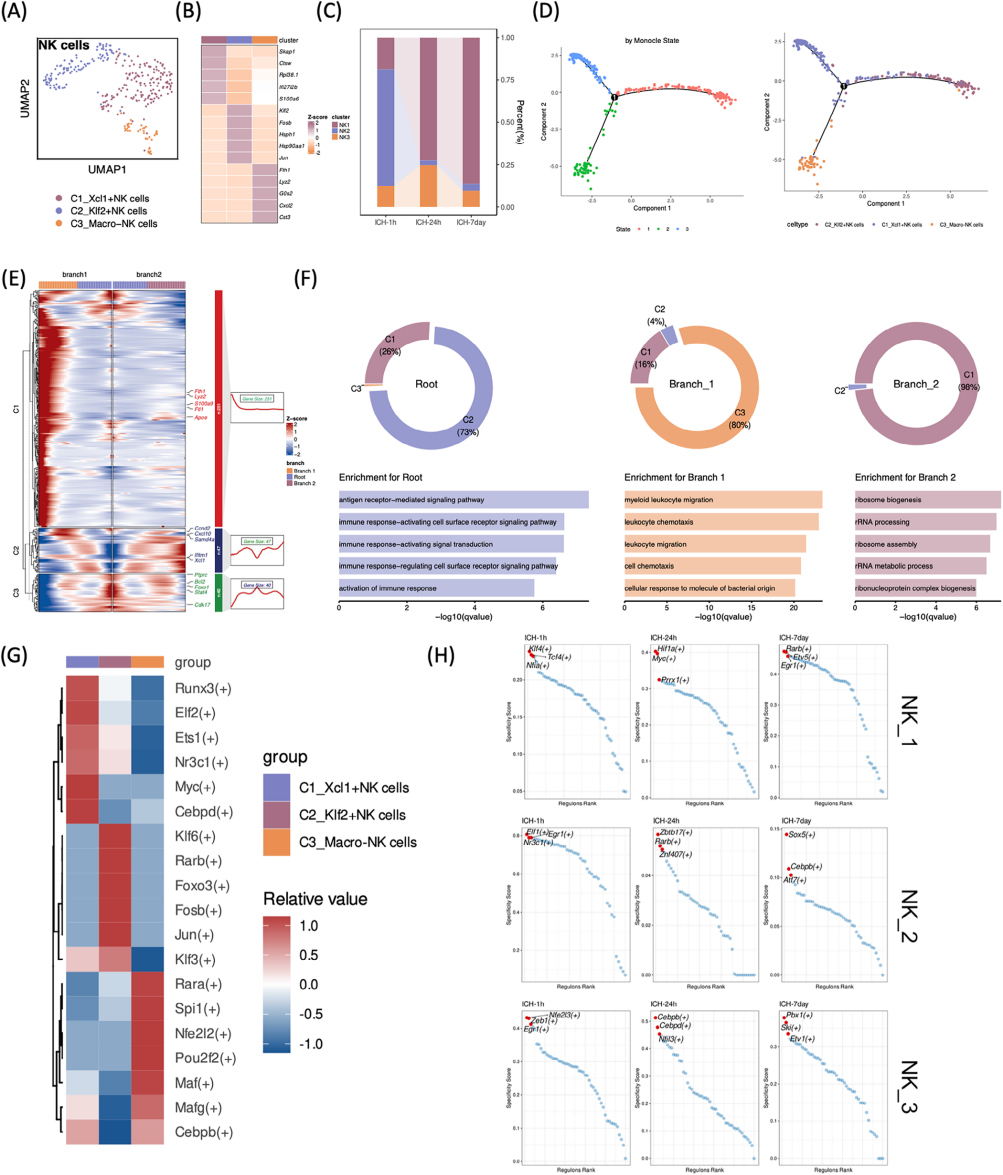
Trajectory of the NK cells post hemorrhagic stroke. (A) UMAP visualization of a total of 313 NK cells in Figure 1B, annotated and colored based on clustering. (B) Heatmap displaying the average expression levels of the top 5 marker genes in each NK‐cell subcluster. (C) Bar plots showing the proportions of the three NK‐cell subclusters at each time point. (D) Pseudotime trajectory trees showing the ordering of NK‐cell clusters marked by Seurat (left) or Monocle (right). (E) BEAM analysis of branch point 1 revealed three clusters of branch‐dependent genes. Heatmap on the left demonstrating the transcriptional changes of these genes from root to branch 1/2. The gene counts and the selected five genes of each gene cluster were shown on the right. The gene expression was *Z*‐score transformed. (F) Pie plots showing the distribution of NK‐cell clusters in each defined Monocle state (Root: State 1; Branch 1: State 2; Branch 2: State 3). (G) Bar plots illustrating the selected top GO‐BP terms related to the three clusters of branch‐dependent genes in (E). (H) Heatmap presenting the average regulon activities in each NK‐cell subcluster, with colors ranging from blue to red indicating relative expression levels, from low to high. (I) Dot plots showing the specialty score of each regulon of NK_1, NK_2, and NK_3 at each time point. The top three regulons with highest specialty scores were annotated.

Three NK cell clusters were identified: C1_Xcl1+ (Skap1, Ctsw, Rpl38.1, Ifi27l2b, S100a6), C2_Klf2+ (Klf2, Fosb, Hsph1, Hsp90aa1, Jun), and C3_Macro− (Fth1 Lyz2 G0s2 Cxcl2 Cst3) (Figure 7A,B). Notably, C2_Klf2+NK cells exhibited abundance during the super‐acute phases of ICH but experienced a sharp decline in subsequent stages, whereas C1_Xcl1+NK cells predominated throughout acute and subacute post‐ICH stages. Interestingly, macrophage‐like NK cells showed an acute increase followed by a subsequent decrease post‐ICH (Figure 7C). Employing pseudotime trajectory trees, we organized NK‐cell clusters identified by Seurat and Monocle to detect ICH‐associated NKs in the homeostatic state and discern differentiated cell states, revealing two distinct branch points (Figure 7D). Monocle analysis along the pseudotime trajectory unveiled three gene clusters with pseudotime‐dependent expression patterns. C2_Klf2+NK cells were predominantly situated at the root state, characterized by elevated Ptprc, Bcl2, Foxo1, Stat4, and Cdk17 expression, indicative of immune response activation. Conversely, C3_Macro−NK cells dominated branch 1, displaying heightened expression of S100a9, Apoe, Fth1, Lyz2, and Ftl1, suggesting enrichment in myeloid leukocyte movement, chemotaxis, and cell chemotaxis. In branch 2, C1_Xcl1+NK cells prevailed and exhibited increased expression of Ccnd2, Cxcl10, Samd4a, Ifitm1, and Xcl1, signifying ribosome‐related biogenesis enrichment (Figure 7E,F). Furthermore, we conducted an analysis of TFs within each cell subtype across stages. Notably, C3_Macro−NK cells exhibited higher expression of TFs Rara, Spi1, Nfe2l2, Pou2f2, Maf, Mafg, and Cebpb. Nfe213, Zeb1, and Egr1 were prevalent during super‐acute stages (1 h) following ICH. Following ICH, C3_Macro−NK cells showed increased expression of Cebpb, Cebpd, and Nfil3 in acute phases (24 h) and Pbx1, Ski, and Etv1 in subacute phases (d7) (Figure 7G,H).

### 2.8 Molecular interaction between myeloid cells and macrophage‐related lymphoid cells within choroid plexus

**FIGURE 8:**
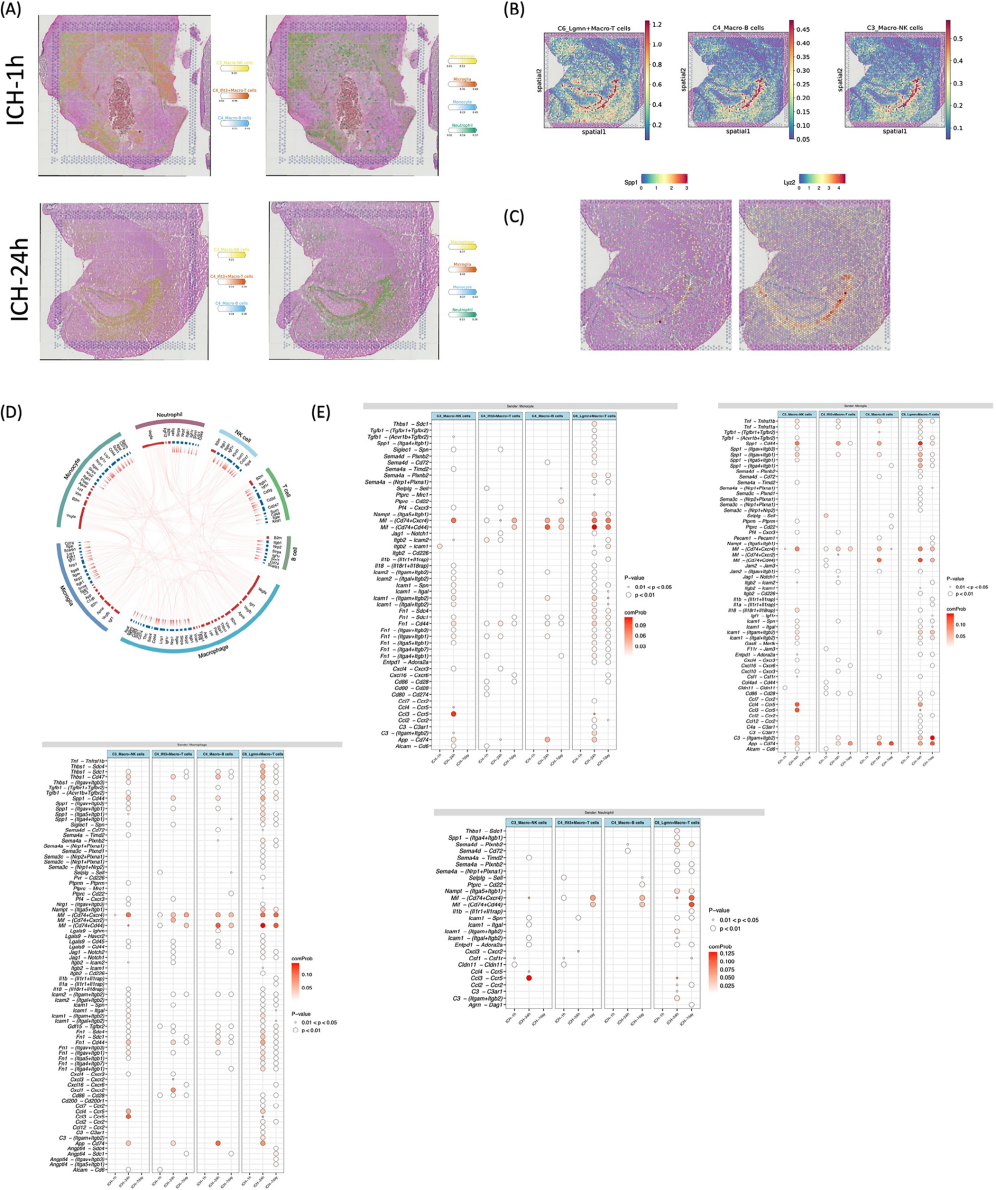
Cell–cell communication among myeloid and macrophage‐related lymphocyte lineages in the posthemorrhagic stroke microenvironment. (A) Spatial colocalization of myeloid and macrophage‐related lymphocyte lineages across regions of rat brains at ICH‐1 h (up) and ICH‐24 h (bottom). (B) Estimated cell abundances (color intensity) of macrophage‐related lymphocyte lineages across regions of rat brains at ICH‐24 h. (C) Spatial plots of Spp1 and Lyz2 expression in histologic sections of ICH‐24 h. (D) Circle diagram illustrating the intercellular receptor ligands among myeloid and lymphocyte lineages. The first track represents each cell type. The second track indicates the ligand–receptor interactions, with red track representing ligands and blue track representing receptors. Each pair of ligand–receptor is connected by a line with an arrowhead, with the top ligands highlighted in red. (E) Dot plots showing the probable ligand–receptor pairs sending from myeloid lineages including monocyte, microglia, macrophage, and neutrophil to lymphocyte lineages including C4_Ifit3+Macro‐T cells, C6_Lgmn+Macro‐T cells, C4_Macro‐B cells, and C3_Macro‐NK cells subsets. The sizes of the dots represent the probability of the ligand–receptor pair.

Elucidating the complex interplay between myeloid cells and macrophage‐related lymphoid cells in the immunological microenvironment is pivotal for deciphering the mechanisms of homeostatic maintenance and inflammatory pathogenesis post‐ICH. Spatial distribution showed that myeloid and macrophage‐related lymphoid enriched in choroid plexus (Figure 8A,B). A distinct subpopulation of T, B, and NK cells exhibited substantial expression of spp1 and lyz2 genes, with their transcripts dispersed in the choroid plexus at 24 h post‐ICH, indicative of intricate intracellular interactions (Figure 8C). Intercellular receptor–ligand dynamics between these two lineages were studied using the iTALK R program, revealing a rich network of broadcasted ligands and cognate receptor communication as depicted in the circos plot (Figure 8D). Employing the CellChat R program, we delved into the complex cell–cell communication network from myeloid to lymphocyte lineages. Intriguingly, Lgmn+Macro‐T cells exhibited heightened interactions with other cell types during the acute phase after ICH. While numerous ligand–receptor pairings were identified, our focus centered on repair‐associated proteins and cytokines such as Chemokine C‐C motif chemokine ligand 3 (Ccl3), macrophage migration inhibitory factor (MIF), semaphorin‐4A, and secreted phosphoprotein 1 (Spp1). Specifically, myeloid cell‐derived Mif and Spp1 ligands displayed increased interaction with Lgmn+Macro‐T cells’ Cd44 and Cd74+Ccr4 receptors throughout acute and subacute post‐ICH stages. Furthermore, myeloid cell‐derived Ccl3 ligands exhibited enhanced interaction with Macro‐NK cells' Ccr5 receptor during the acute phase post‐ICH (Figure 8E).

## 3 DISCUSSION

The evolving comprehension of the brain's immunological landscape during stroke pathogenesis entails the intricate interplay between myeloid and lymphocytic cells, showcasing dynamic fluctuations in infiltrating immune cell proportions at distinct temporal intervals.^2^, ^13^ In contrast to conventional methodologies reliant on flow cytometry and limited markers, our study harnessed high‐dimensional, single‐cell resolution analysis, enabling meticulous ST profiling of immune cell dynamics following ICH across different phases (1 h, 24 h, 7d). Our investigation unveils several pivotal findings: (1) *Cell population dynamics*: In the early acute phase (24 h) post‐ICH, myeloid cells predominated as the key cell population, gradually yielding ground to increased lymphocyte proportions during the ensuing subacute phase (d7). ST analysis highlighted immune cell enrichment in the choroid plexus at the 24‐h mark post‐ICH. (2) *Distinct myeloid subtypes*: We identified more types of MG: anti‐inflammatory, homeostatic, proliferating, myelin‐accumulating, proinflammation, nonlipid‐phagocytosing, and lipid‐droplet‐formation. Monocyte/macrophages that are relatively low in development at the beginning of the process are almost exclusively clustered in the choroid plexus and choroid plexus regions, whereas more maturely developed cells are all migrating distally to the striatum and cerebral cortex. (3) *Lymphocyte dynamics*: A subgroup of lymphocytes expressing heightened macrophage‐related markers (spp1 and lyz2) manifested transiently during the acute phase (24 h), tapering in the subsequent subacute phase (d7) post‐ICH. Additionally, robust associations between myeloid cells and macrophage‐related lymphocytes around the choroid plexus, particularly via the spp1–cd44 pathway during the acute phase, were observed.

Our study underscores the dominance of myeloid cells following ICH, consistently outnumbering lymphoid cells across the studied stages, aligning with prior research.^14^ While microglial subpopulations and gene expression patterns have been explored in ischemic stroke,^15^ our single‐cell investigation marks a pioneering effort in hemorrhagic stroke research. Specifically, our study sheds unique light on microglial spatial characterization after ICH. Our comprehensive microglial analysis delineated seven subgroups executing homeostatic, proinflammatory, and anti‐inflammatory functions, encapsulating the intricate immunological response postbrain damage. Notably, Mg0 and Mg4 subsets exhibited notably diverse transcriptomic profiles. The heightened expression of Msr1, Spp1, and Lcn2 in the Mg4 subset suggests proinflammatory functions and potential therapeutic avenues. After inflammation, infection, or damage, the CNS synthesizes and releases LCN2.^16^ LCN2 may regulate macrophage morphologies and generate chemokines to engage in inflammatory responses.^17^ After stroke, SPP1+ MG caused pontine infarction axonal demyelination.^18^ MSR1 aids phagocytosis. Moreover, the predominant Mg0 subgroup at subacute stages (7d) after stroke expressed greater Nav3 and Dock4, pivotal for axon outgrowth, guidance,^19^ and cellular protection against oxidative stress,^20^ implying a neuroprotective potential. Immunofluorescence analysis 24 h after stroke corroborated Msr1 and LCN2 expression. ST underscored the significant presence of a microglial subpopulation in the choroid plexus, with marked expression of lcn2 and msr1, indicative of intricate biological activity.

Neutrophils, pivotal in innate immunity and poststroke neuroinflammation, assume specialized roles across various post‐ICH phases,^21^, ^22^ including a Neu_4 subset expressing genes (Orm1, Mt3, Ifitm3, Cfb) implicated in oxidative stress suppression, potentially beneficial in later stages. Orosomucoid 1 (Orm1), an adipocytokine family, protects adipose tissue against inflammation.^23^ Metallothionein‐3 (MT3) regulates physiological processes and protects cells from oxidative stress by maintaining copper and zinc homeostasis.^24^ TTR also plays a neuroprotective role.^7^ The infiltration of Neu_4 into the brain during later post‐ICH phases might signify a beneficial response. Furthermore, there is minimal alteration in spatial distribution amongst neutrophil subsets. Monocytes/macrophages, integral to the immunological response, exhibit distinct subsets that regulate inflammation, facilitate tissue remodeling, and orchestrate immune responses.^25^ Various monocyte/macrophage subsets were observed at different post‐ICH phases. The gene expression analysis of these subgroups unveiled their involvement in inflammation control, tissue remodeling, and immune response coordination. Significantly, we observe that monocyte/macrophages, which exhibit relatively lower developmental stages at the onset of the process, predominantly cluster within the choroid plexus and choroid plexus regions, while more mature cell populations migrate distally to the striatum and cerebral cortex.

Following ICH, subpopulations of T, B, and NK lymphocytes expressing macrophage‐associated markers (spp1 and lyz2) exhibit a peak during the acute phase, followed by a decline in the subacute phase, indicating their potential involvement in ICH pathogenesis. ST unveiled potential crosstalk between myeloid cells and macrophage‐related lymphoid cells within the choroid plexus, aligning with recent literature highlighting the choroid plexus's crucial role in CSF production and its regulation, thereby impacting lymphoid system function.^26^ The orchestrated circulation of CSF facilitates the clearance of blood and its derivatives from ICH sites, thereby contributing to brain repair processes.^27^, ^28^ The observation highlights the multifaceted role of the choroid plexus in sustaining CNS homeostasis and underscores its potential in mediating posthemorrhagic brain repair mechanisms. Intriguingly, cell trajectory analysis unveiled that all macrophage‐associated lymphocytes predominantly emerge at developmental endpoints, particularly within this branch. Predominantly found TFs such as S100a9, Apoe, Fth1, Lyz2, and Ftl1 in this branch suggest a potential contribution of macrophage‐associated lymphocytes to ICH. Subsequently, we delved into the intricate interaction between myeloid cells and macrophage‐related lymphoid cells, revealing active crosstalk between Lgmn+Macro‐T cells and myeloid cell subsets mediated via the spp1–cd44 pathway.

While our study offers significant insights, it is important to acknowledge certain limitations. First, our investigation into immune cell alterations was restricted to discrete post‐ICH phases, potentially overlooking nuanced changes occurring outside these defined timeframes. Moreover, our analyses were conducted exclusively in adult mice, and it is conceivable that immune profiles may vary across different age groups, necessitating caution in extrapolating these findings to other populations. Additionally, we did consider scMLnet,^29^ a great tool analyzing intercellular and intracellular signaling networks, our analysis did not identify any significant ligand–receptor pairs in our scRNA‐seq data.

## 4 CONCLUSIONS

Our study provides a comprehensive analysis of the immune microenvironment's dynamic evolution following ICH. The elucidation of immune cell dynamics and their intricate interactions across injury and recovery phases yields critical insights into ICH pathophysiology. This knowledge may inform novel therapeutic approaches that harness the immune response to promote recovery and mitigate damage post‐ICH.

## 5 MATERIALS AND METHODS

### 5.1 ScRNA sequencing and ST

Animals were euthanized at 1 h, 24 h, or 7 days after ICH. Hemorrhagic hemispheres were harvested without meninges, the olfactory bulb, or cerebellum. Hemorrhagic hemispheres at ICH‐1 h, ICH‐24 h, and ICH‐7day (three replicates each) were sent for scRNA‐seq while hemorrhagic hemispheres at ICH‐1 h and ICH‐24 h (two replicates each) were sent for ST.

For scRNA‐seq, brain tissue was homogenized using the Adult Brain Dissociation Kit (T) and a gentle MACS dissociator with heaters (Miltenyi Biotec), adhering to the manufacturer's guidelines. The resulting suspension was filtered through a 70‐µm cell strainer (Thermo Fisher Scientific) to remove debris, and cell viability was assessed using the Cellometer Auto 2000 system (Nexcelom). Samples with >85% viability were processed for scRNA‐seq library preparation. Live single cells were encapsulated into Gel Beads in Emulsion (GEMs) using the Chromium platform (10× Genomics) and Single Cell 3′ v2 chemistry. The GEMs, each containing a single cell and a gel bead, were prepared with a >1000‐fold excess of partitions to ensure single‐cell encapsulation. Following mRNA capture and barcoding, the emulsion was processed off‐instrument for reverse transcription. The cDNA was then fragmented, amplified, and prepared according to the 10× protocol. The libraries were purified, quantified, and sequenced on an Illumina NovaSeq 6000 platform, targeting a minimum of 100,000 reads per cell with a paired‐end 120 bp strategy.

For ST, fresh brain tissues were sectioned into fragments, embedded in optimal cutting temperature compound (Sakura Finetek, Torrance, USA), and flash‐frozen on dry ice. Cryosections of 10‐µm thickness were prepared using a cryostat, and these were mounted onto ST expression slides designed to accommodate an 8 × 8 mm array with spatially barcoded spots. The tissue sections were dehydrated with isopropanol and stained with hematoxylin and eosin. Bright‐field imaging was performed using a Pannoramic MIDI FL whole slide scanner (3DHISTECH) at 20× magnification. Pathological examination and histological annotation were conducted by two expert pathologists, followed by manual annotation to delineate distinct regions. Slides for library preparation were sourced from the ST team. Each spot on the slide, with a diameter of 55 µm and spaced 100 µm apart, covered an area of 6.5 × 6.5 mm^2^. A total of four capture zones per slide, each with approximately 5000 gene expression spots, were utilized. Tissue processing was carried out using the Visium Spatial Gene Expression Reagent Kits (10× Genomics, Pleasanton, CA), following the standard protocol. Permeabilization was performed with an enzyme at 37°C, followed by washing and reverse transcription using a master mix for cDNA synthesis. Full‐length cDNA, incorporating spatial barcodes, was synthesized from polyadenylated mRNAs. The first‐strand synthesis was halted using potassium hydroxide, neutralized, and second‐strand synthesis was initiated. This process facilitated the incorporation of spatial barcodes and amplification of full‐length cDNA via PCR, yielding sufficient material for library construction. Library construction adhered to the Visium Spatial Gene Expression User Guide (CG000239, Rev B; 10× Genomics). The constructed libraries were sequenced on Illumina NovaSeq 6000 Systems using a paired‐end 150 bp strategy, targeting a minimum of 100,000 reads per spot. Both scRNA‐seq and ST analyses were conducted by CapitalBio Technology (Beijing, China).

### 5.2 scRNA‐seq data analysis

#### 5.2.1 scRNA‐seq data preprocessing and quality control

All scRNA‐seq sample gene count matrices underwent processing using Seurat (v. 4.4.0)^30^, ^31^, ^32^, ^33^ within the R (v. 4.3.1) environment, aimed at quality control and subsequent analyses. Initially, cells of inferior quality were excluded based on the quantity of identifiable genes. Subsequently, gene count matrices were normalized against total cellular read counts employing the “LogNormalize” function in the Seurat package. Following normalization, the data were subjected to scaling and centering to facilitate further analysis.

#### 5.2.2 Cell‐clustering, annotation, and doublet removal

For dimensionality reduction, we employed the “FindVariableFeatures” function in Seurat, utilizing the “vst” method to identify the 2000 most variable genes based on their highest standardized variance. These genes were subjected to PCA. Subsequently, “FindNeighbors” and “FindClusters” functions were applied to delineate cell clusters. A shared nearest‐neighbor (SNN) graph was constructed using the top 30 PCs, calculating the neighborhood overlap between each cell and its nearest neighbors. Clustering was performed using an SNN modularity optimization‐based algorithm. To visualize distinct cell clusters, UMAP was executed on the top 30 PCs via the “RunUMAP” function. For cell cluster annotation, DEGs were identified using the “FindMarkers” function. Clusters were annotated comprehensively using three approaches: (1) SingleR automatic annotation, (2) CellMarker online portal, and (3) classical markers collection by literature reviewing (Table S1). Next, normalization, dimensionality reduction, batch effects mitigation with Harmony (v.1.2.0). Doublets were identified by the Doublefinder (v. 2.0.4) and removed.

#### 5.2.3 Functional enrichment analysis

Functional enrichment analysis was performed with the clusterProfiler R package (v.4.8.1).^34^ The entire rat genome served as the reference background for enrichment analysis. Following the identification of a list of DEGs, Gene Ontology (GO) Biological Processes enrichment analysis was conducted, employing a minimum gene count threshold of three.

#### 5.2.4 Trajectory and pseudo‐time ordering analysis

The trajectory and pseudotime ordering analysis of T cells, B cells, and NK cells were performed by the Monocle R package (v.2.14.0).^35^, ^36^, ^37^ Briefly, the ordering genes were defined as the top 2000 previously identified highly variable genes. The dimensionality of the data was reduced by the “DDRTree” method of the *reduceDimension* function. The trajectory was then developed using the *OrderCells* and visualized by the *Plot_Cell_Trajectory* functions. After the starting point of the cellular trajectory was chosen, the pseudotemporal order of cells was generated. To identify branch‐dependent genes in the trajectory, branch expression analysis modeling (BEAM) was employed by the *BEAM* function. Another method for inferring cell lineages from scRNA‐seq data, Slingshot (v.2.6.0) R package^38^ was applied for trajectory analysis of the macrophages and monocytes. Two naïve subtypes, Macro_1 and Mono_2, were selected as two starting points to infer multiple potential trajectories.

CytoTRACE (v.0.3.3)^39^ was applied to predict the differentiation scores of neutrophils. It is a computational framework reconstructing the differentiation state of cells using scRNA‐seq data. Briefly, a K‐nearest neighbors (KNN) graph was constructed to represent the connections between cells, without considering the directionality of these relationships. The CytoTRACE algorithm was employed to determine the optimal chronological order of cells. Subsequently, the KNN graph, as well as the previously determined chronological information, were used to generate a transfer matrix. The resulting transfer matrix was visualized using a UMAP scatter plot.

#### 5.2.5 Orthologous genes transformation

The homologene R package (v.1.4.68) was used to convert the mouse genes into human genes. Briefly, the *homologene* function receives a list of Rattus norvegicus genes (inTax: 10116) and returns a data frame including the genes and their corresponding homologues (outTax: 9606).

#### 5.2.6 Cell–cell interaction analysis

After orthologous genes transformation, the iTALK R package (v.0.1.1)^40^ was applied to elucidate the intercellular receptor–ligand changes between the myeloid and lymphocyte lineages. Briefly, the *rawParse* function was employed to get top 25 expressed genes from the scRNA‐seq data. The *FindLR* function loaded the highly expressed genes to infer significant interactions based on the ligand–receptor database implemented in this package. The *LRPlot* function was utilized to visualize the significant ligand–receptor interactions.

The CellChat R package (v.1.6.1)^41^ was applied to infer and analyze cell–cell communication from myeloid lineage to lymphocyte lineage. Briefly, the normalized scRNA‐seq data were loaded into CellChat and preprocessed by *identifyOverExpressedGenes* and *identifyOverExpressedInteractions* functions with default parameters according to the official workflow. To identify potential cell–cell communication networks, the core functions *computeCommunProb*, *computeCommunProbPathway* and *aggregateNet* were applied with default parameters. The strengths of predicted ligand–receptor pairs were then visualized using ggplot2 R package (v.3.4.3).

### 5.3 ST data analysis

Following the processing of fastq files through alignment, filtering, barcoding, and Unique Molecular Identifier counting with SpaceRanger (version 2.0.1; 10× Genomics), an average of 2787 distinct genes per spot was identified. The output files were subsequently imported into the Seurat package (version 4.3.0)^30^, ^31^, ^32^, ^33^ in R for normalization using the SCTransform algorithm. Clustering of ST spots was conducted using a method analogous to that applied in scRNA‐seq data analysis, with the top 20 PCs from PCA employed to determine neighbor relationships. Genes differentially expressed within a ST cluster, in contrast to all other clusters, were identified with a *p* value threshold of less than 0.01 from the Wilcoxon rank sum test and an average log fold‐change greater than a defined threshold. The CARD version,^42^ a reference‐based deconvolution method, was utilized to estimate cell type composition within ST sections, leveraging cell type‐specific expression profiles derived from a reference scRNA‐seq dataset.

### 5.4 Determination of cell type enrichment/depletion by MIA

To integrate single‐cell expression profiles with spatial transcriptome data, MIA^43^ was used to delineate sets of cell type‐specific (scRNA‐seq identified cell types) and tissue region‐specific genes (histological features) and then determining whether their overlap is higher (enrichment) or lower (depletion) than expected by chance.

Detailed materials and methods can be found in Supplementary Information Additional File 1.

## AUTHOR CONTRIBUTIONS

Lingui Gu and Hualin Chen drafted the work. Yihao Chen and Jianbo Chang collect the specimen. Mingjiang Sun and Qinglei Shi analyses the data. Junji Wei and Wenbin Ma drew the figures. Xinjie Bao and Renzhi Wang devised the work. All authors read and approved the final manuscript.

## CONFLICT OF INTEREST STATEMENT

The authors have no conflict of interest to declare

## ETHICS STATEMENT

Our experimental protocols were approved by the Animal Ethics Committee of the Chinese Academy of Medical Sciences and Peking Union Medical College (XHDW‐2022‐085). All animal studies complied with the National Institutes of Health guide for the care and use of Laboratory Animals and the ARRIVE (Animal Research: Reporting In Vivo Experiments) guidelines and received approval from the Institutional Animal Care and Use Committee of the Chinese Academy of Medical Sciences and Peking Union Medical College.

## Supporting information

Supporting Information

## Data Availability

The raw sequence data reported in this paper have been deposited in the Genome Sequence Archive (Genomics, Proteomics & Bioinformatics 2021)^44^ in National Genomics Data Center (Nucleic Acids Res 2022),^45^ China National Center for Bioinformation/Beijing Institute of Genomics, Chinese Academy of Sciences (*GSA: CRA016497*) and (*GSA: CRA016522*) that are publicly accessible at https://ngdc.cncb.ac.cn/gsa.

## References

[1] Johnson W , Onuma O , Owolabi M , Sachdev S . Stroke: a global response is needed. Bull World Health Organ. 2016;94(9):634‐634A. 27708464 10.2471/BLT.16.181636 PMC5034645

[2] Liu R , Song P , Gu X , et al. Comprehensive landscape of immune infiltration and aberrant pathway activation in ischemic stroke. Front Immunol. 2021;12:766724. 35140708 10.3389/fimmu.2021.766724 PMC8818702

[3] Iadecola C , Buckwalter MS , Anrather J . Immune responses to stroke: mechanisms, modulation, and therapeutic potential. J Clin Invest. 2020;130(6):2777‐2788. 32391806 10.1172/JCI135530 PMC7260029

[4] Allen WE , Blosser TR , Sullivan ZA , Dulac C , Zhuang X . Molecular and spatial signatures of mouse brain aging at single‐cell resolution. Cell. 2023;186(1):194‐208. e118. 36580914 10.1016/j.cell.2022.12.010 PMC10024607

[5] Chua RL , Lukassen S , Trump S , et al. COVID‐19 severity correlates with airway epithelium‐immune cell interactions identified by single‐cell analysis. Nat Biotechnol. 2020;38(8):970‐979. 32591762 10.1038/s41587-020-0602-4

[6] Wagenhauser MU , Mulorz J , Krott KJ , et al. Crosstalk of platelets with macrophages and fibroblasts aggravates inflammation, aortic wall stiffening, and osteopontin release in abdominal aortic aneurysm. Cardiovasc Res. 2024;120(4):417‐432. 37976180 10.1093/cvr/cvad168

[7] Wieczorek E , Ozyhar A . Transthyretin: from structural stability to osteoarticular and cardiovascular diseases. Cells. 2021;10(7):1768. 34359938 10.3390/cells10071768 PMC8307983

[8] Qi J , Sun H , Zhang Y , et al. Single‐cell and spatial analysis reveal interaction of FAP(+) fibroblasts and SPP1(+) macrophages in colorectal cancer. Nat Commun. 2022;13(1):1742. 35365629 10.1038/s41467-022-29366-6 PMC8976074

[9] Zhu J , Fan Y , Xiong Y , et al. Delineating the dynamic evolution from preneoplasia to invasive lung adenocarcinoma by integrating single‐cell RNA sequencing and spatial transcriptomics. Exp Mol Med. 2022;54(11):2060‐2076. 36434043 10.1038/s12276-022-00896-9 PMC9722784

[10] Gerganova G , Riddell A , Miller AA . CNS border‐associated macrophages in the homeostatic and ischaemic brain. Pharmacol Ther. 2022;240:108220. 35667516 10.1016/j.pharmthera.2022.108220

[11] Silvin A , Uderhardt S , Piot C , et al. Dual ontogeny of disease‐associated microglia and disease inflammatory macrophages in aging and neurodegeneration. Immunity. 2022;55(8):1448‐1465. e1446. 35931085 10.1016/j.immuni.2022.07.004

[12] Morath A , Schamel WW . alphabeta and gammadelta T cell receptors: similar but different. J Leukoc Biol. 2020;107(6):1045‐1055. 31994778 10.1002/JLB.2MR1219-233R

[13] Goods BA , Askenase MH , Markarian E , et al. Leukocyte dynamics after intracerebral hemorrhage in a living patient reveal rapid adaptations to tissue milieu. JCI Insight. 2021;6(6):e145857. 33749664 10.1172/jci.insight.145857 PMC8026179

[14] Mei S , Shao Y , Fang Y , et al. The changes of leukocytes in brain and blood after intracerebral hemorrhage. Front Immunol. 2021;12:617163. 33659003 10.3389/fimmu.2021.617163 PMC7917117

[15] Morrison HW , Filosa JA . A quantitative spatiotemporal analysis of microglia morphology during ischemic stroke and reperfusion. J Neuroinflammation. 2013;10:4. 23311642 10.1186/1742-2094-10-4 PMC3570327

[16] Jha MK , Jeon S , Jin M , et al. The pivotal role played by lipocalin‐2 in chronic inflammatory pain. Exp Neurol. 2014;254:41‐53. 24440229 10.1016/j.expneurol.2014.01.009

[17] Ni W , Zheng M , Xi G , Keep RF , Hua Y . Role of lipocalin‐2 in brain injury after intracerebral hemorrhage. J Cereb Blood Flow Metab. 2015;35(9):1454‐1461. 25853903 10.1038/jcbfm.2015.52 PMC4640334

[18] Luo M , Qiu Z , Tang X , et al. Inhibiting cyclin B1‐treated pontine infarction by suppressing proliferation of SPP1+ microglia. Mol Neurobiol. 2023;60(4):1782‐1796. 36572839 10.1007/s12035-022-03183-w

[19] Uzdensky A , Demyanenko S , Fedorenko G , Lapteva T , Fedorenko A . Protein profile and morphological alterations in penumbra after focal photothrombotic infarction in the rat cerebral cortex. Mol Neurobiol. 2017;54(6):4172‐4188. 27324898 10.1007/s12035-016-9964-5

[20] Li S , Chen S , Wang Y , et al. Direct targeting of DOCK4 by miRNA‐181d in oxygen‐glucose deprivation/reoxygenation‐mediated neuronal injury. Lipids Health Dis. 2023;22(1):34. 36882763 10.1186/s12944-023-01794-3 PMC9990210

[21] Garcia‐Culebras A , Duran‐Laforet V , Pena‐Martinez C , et al. Myeloid cells as therapeutic targets in neuroinflammation after stroke: specific roles of neutrophils and neutrophil‐platelet interactions. J Cereb Blood Flow Metab. 2018;38(12):2150‐2164. 30129391 10.1177/0271678X18795789 PMC6282223

[22] Reichardt LF . Neurotrophin‐regulated signalling pathways. Philos Trans R Soc Lond B Biol Sci. 2006;361(1473):1545‐1564. 16939974 10.1098/rstb.2006.1894 PMC1664664

[23] Zhou B , Luo Y , Ji N , Hu C , Lu Y . Orosomucoid 2 maintains hepatic lipid homeostasis through suppression of de novo lipogenesis. Nat Metab. 2022;4(9):1185‐1201. 36050503 10.1038/s42255-022-00627-4

[24] Howells C , West AK , Chung RS . Neuronal growth‐inhibitory factor (metallothionein‐3): evaluation of the biological function of growth‐inhibitory factor in the injured and neurodegenerative brain. FEBS J. 2010;277(14):2931‐2939. 20561053 10.1111/j.1742-4658.2010.07718.x

[25] Kim E , Cho S . Microglia and monocyte‐derived macrophages in stroke. Neurotherapeutics. 2016;13(4):702‐718. 27485238 10.1007/s13311-016-0463-1 PMC5081116

[26] Fang Y , Huang L , Wang X , et al. A new perspective on cerebrospinal fluid dynamics after subarachnoid hemorrhage: from normal physiology to pathophysiological changes. J Cereb Blood Flow Metab. 2022;42(4):543‐558. 34806932 10.1177/0271678X211045748 PMC9051143

[27] Solar P , Klusakova I , Jancalek R , Dubovy P , Joukal M . Subarachnoid hemorrhage induces dynamic immune cell reactions in the choroid plexus. Front Cell Neurosci. 2020;14:18. 32116563 10.3389/fncel.2020.00018 PMC7026251

[28] Zhang Z , Tan Q , Guo P , et al. NLRP3 inflammasome‐mediated choroid plexus hypersecretion contributes to hydrocephalus after intraventricular hemorrhage via phosphorylated NKCC1 channels. J Neuroinflammation. 2022;19(1):163. 35729645 10.1186/s12974-022-02530-x PMC9210649

[29] Cheng J , Zhang J , Wu Z , Sun X . Inferring microenvironmental regulation of gene expression from single‐cell RNA sequencing data using scMLnet with an application to COVID‐19. Brief Bioinform. 2021;22(2):988‐1005. 33341869 10.1093/bib/bbaa327 PMC7799217

[30] Hao Y , Hao S , Andersen‐Nissen E , et al. Integrated analysis of multimodal single‐cell data. Cell. 2021;184(13):3573‐3587. e3529. 34062119 10.1016/j.cell.2021.04.048 PMC8238499

[31] Stuart T , Butler A , Hoffman P , et al. Comprehensive integration of single‐cell data. Cell. 2019;177(7):1888‐1902. e1821. 31178118 10.1016/j.cell.2019.05.031 PMC6687398

[32] Butler A , Hoffman P , Smibert P , Papalexi E , Satija R . Integrating single‐cell transcriptomic data across different conditions, technologies, and species. Nat Biotechnol. 2018;36(5):411‐420. 29608179 10.1038/nbt.4096 PMC6700744

[33] Satija R , Farrell JA , Gennert D , Schier AF , Regev A . Spatial reconstruction of single‐cell gene expression data. Nat Biotechnol. 2015;33(5):495‐502. 25867923 10.1038/nbt.3192 PMC4430369

[34] Wu T , Hu E , Xu S , et al. clusterProfiler 4.0: a universal enrichment tool for interpreting omics data. Innovation (Camb). 2021;2(3):100141. 34557778 10.1016/j.xinn.2021.100141 PMC8454663

[35] Trapnell C , Cacchiarelli D , Grimsby J , et al. The dynamics and regulators of cell fate decisions are revealed by pseudotemporal ordering of single cells. Nat Biotechnol. 2014;32(4):381‐386. 24658644 10.1038/nbt.2859 PMC4122333

[36] Qiu X , Hill A , Packer J , Lin D , Ma YA , Trapnell C . Single‐cell mRNA quantification and differential analysis with Census. Nat Methods. 2017;14(3):309‐315. 28114287 10.1038/nmeth.4150 PMC5330805

[37] Qiu X , Mao Q , Tang Y , et al. Reversed graph embedding resolves complex single‐cell trajectories. Nat Methods. 2017;14(10):979‐982. 28825705 10.1038/nmeth.4402 PMC5764547

[38] Street K , Risso D , Fletcher RB , et al. Slingshot: cell lineage and pseudotime inference for single‐cell transcriptomics. Bmc Genomics [Electronic Resource]. 2018;19(1):477. 29914354 10.1186/s12864-018-4772-0 PMC6007078

[39] Gulati GS , Sikandar SS , Wesche DJ , et al. Single‐cell transcriptional diversity is a hallmark of developmental potential. Science. 2020;367(6476):405‐411. 31974247 10.1126/science.aax0249 PMC7694873

[40] Wang Y , Wang R , Zhang S , et al. iTALK: an R package to characterize and illustrate intercellular communication. Biorxiv. 2019:507871.

[41] Jin S , Guerrero‐Juarez CF , Zhang L , et al. Inference and analysis of cell‐cell communication using CellChat. Nat Commun. 2021;12(1):1088. 33597522 10.1038/s41467-021-21246-9 PMC7889871

[42] Ma Y , Zhou X . Spatially informed cell‐type deconvolution for spatial transcriptomics. Nat Biotechnol. 2022;40(9):1349‐1359. 35501392 10.1038/s41587-022-01273-7 PMC9464662

[43] Moncada R , Barkley D , Wagner F , et al. Integrating microarray‐based spatial transcriptomics and single‐cell RNA‐seq reveals tissue architecture in pancreatic ductal adenocarcinomas. Nat Biotechnol. 2020;38(3):333‐342. 31932730 10.1038/s41587-019-0392-8

[44] Chen T , Chen X , Zhang S , et al. The genome sequence archive family: toward explosive data growth and diverse data types. Genomics Proteomics Bioinformatics. 2021;19(4):578‐583. 34400360 10.1016/j.gpb.2021.08.001 PMC9039563

[45] Members C‐N, Partners . Database Resources of the National Genomics Data Center, China National Center for Bioinformation in 2022. Nucleic Acids Res. 2022;50(D1):D27‐D38. 34718731 10.1093/nar/gkab951 PMC8728233

