# Supplementary material for: Unraveling dynamic immunological landscapes in intracerebral hemorrhage: insights from single‐cell and spatial transcriptomic profiling
Source: MedComm (2020). 2024 Jul 10;5(7):e635. doi: 10.1002/mco2.635 (PMC11233862; doi:10.1002/mco2.635)
Supplement: Supplementary file 1 — Supporting Information [file MCO2-5-e635-s001.docx]

**Additional file 1**

**Unraveling Dynamic Immunological Landscapes in Intracerebral Hemorrhage: Insights from Single-Cell and Spatial Transcriptomic Profiling**

Lingui Gu^1#^, Hualin Chen^1#^, Mingjiang Sun^2#^, Yihao Chen^1^, Qinglei Shi^3^, Jianbo Chang^1^, Junji Wei^1^, Wenbin Ma^1^, Xinjie Bao^1,4*^, Renzhi Wang^1,5*^

^#^These authors contributed equally to this work.

^1^ Peking Union Medical College Hospital, Chinese Academy of Medical Sciences and Peking Union Medical College, Beijing 100730, China

^2^ Beijing Neurosurgical Institute, Beijing Tiantan Hospital, Capital Medical University. Beijing, 100070, China.

^3^ Research Institute of Big Data, Chinese University of Hong Kong (Shenzhen) School of Medicine, Shenzhen, China

^4^ State Key Laboratory of Common Mechanism Research for Major Diseases, Beijing, China

^5^ School of Medicine, The Chinese University of Hong Kong, Shenzhen, Guangdong, 518172, P. R. China

***Corresponding author**

Xinjie Bao, MD, Department of Neurosurgery, Peking Union Medical College Hospital, Chinese Academy of Medical Sciences and Peking Union Medical College, Beijing, China, 100730; Tel: +86-10-69152530; E-mail: [baoxinjie1@pumch.cn](mailto:baoxinjie1@pumch.cn)

Renzhi Wang, MD, School of Medicine, The Chinese University of Hong Kong, Shenzhen, Guangdong, 518172, P.R. China; Tel: +86-18612671601; E-mail: [wangrz@126.com](mailto:wangrz@126.com)

**Materials and methods**

**Magnetic resonance imaging**

Animals were secured on a detachable stage, immobilized with paper towels and plastic wrap, and insulated to maintain body warmth. Each rat underwent intubation and was maintained under anesthesia with a 3% isoflurane-oxygen mixture at a 7:3 ratio, facilitated by a rodent ventilator from Harvard Apparatus, Holliston, MA, USA. Subsequently, the animals were positioned within a custom-designed, shielded 8-leg high-pass birdcage radiofrequency (RF) coil, 65 mm in diameter, which served for RF pulse transmission and signal reception. The MRI experiments were conducted using a 7T Oxford 300/150 horizontal bore magnet with a 150 mm inner bore (Oxford Instruments Limited, UK) and an MRRS console (MR Research Systems Ltd., Surrey, UK). The system is equipped with an actively shielded gradient coil, 90 mm in inner diameter and capable of a maximum gradient strength of 750 mT/m (Model BFG-150/90-S, Resonance Research Inc., Billerica, MA). The gradient coil is driven by Techron series 7700 linear gradient amplifiers, which have a maximum slew rate of 150 T/m/s. Preliminary tests using T1- and T2-weighted imaging were conducted to optimize settings for in vivo imaging of the rodent prostate.

**Immunofluorescence staining**

Double fluorescence staining was carried out as previously mentioned {Huang, 2015 #258}{Huang, 2015 #259}^15^. The mice were given a deep anaesthetic before having 20 ml of ice-cold PBS and 20 ml of 4% paraformaldehyde transcardially infused into them 24 hours after the ICH. The whole brain was removed and preserved in 4% paraformaldehyde for a further 24 hours. After that, the brain was dehydrated in solutions of 20% and 30% sucrose. The brain was sectioned into 10-m-thick coronal slices using a cryostat (CM1860; Leica Microsystems, Germany) after being frozen at 25 °C. The brain sections were subjected to double immunohistochemistry staining by being incubated at 4 °C overnight with primary antibodies against anti-ionized calcium-binding adaptor molecule 1 (Iba-1, 1:200, Wako, Japan), anti-LCN2 (1:200, Abclonal, China), anti-MSR1 (1:100, Novus, USA), anti-Peroxiredoxin 5 (Prdx5, 1:100, Abclonal, China), and anti-Galectin 3 (Lgals3, 1:100, abcam), anti-CD68 (1:100, Novus, USA), anti-ly6c (1:100, abcam, USA). The slices were seen and captured using a fluorescent microscope (U-HGLGPS, OLYMPUS, Japan) after being treated with the appropriate secondary antibody (1:200, Bioss, China) at 37 °C for 1 h. CellSens Standard software was used to analyse microphotographs.

**Figure.S1**

**Figure.S1** Volcano plot showing the all classical markers for each cell set.

**Figure.S2**


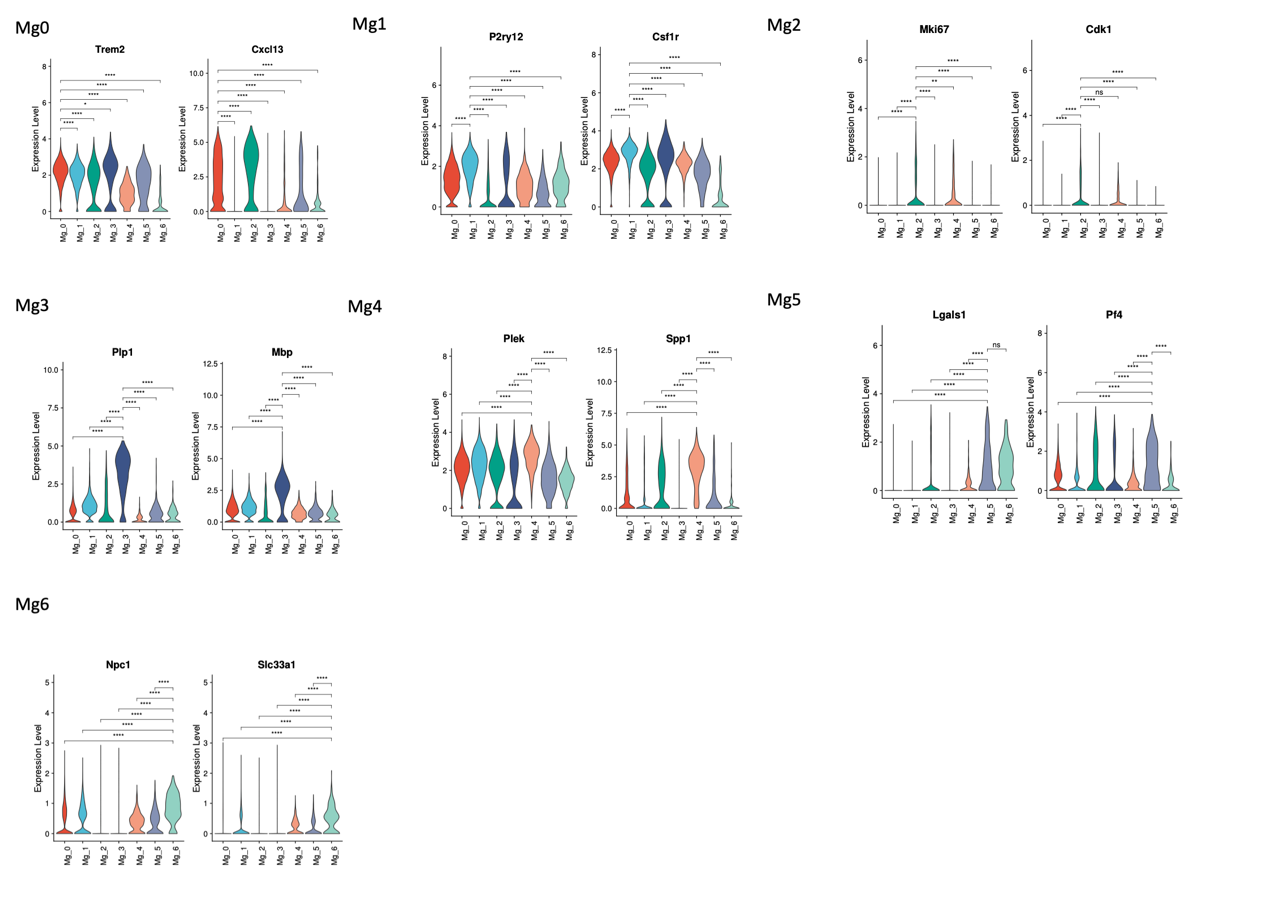


**Figure.S2.**Volcano plot showing the all classical markers for microglia subtypes.

**Figure.S3**


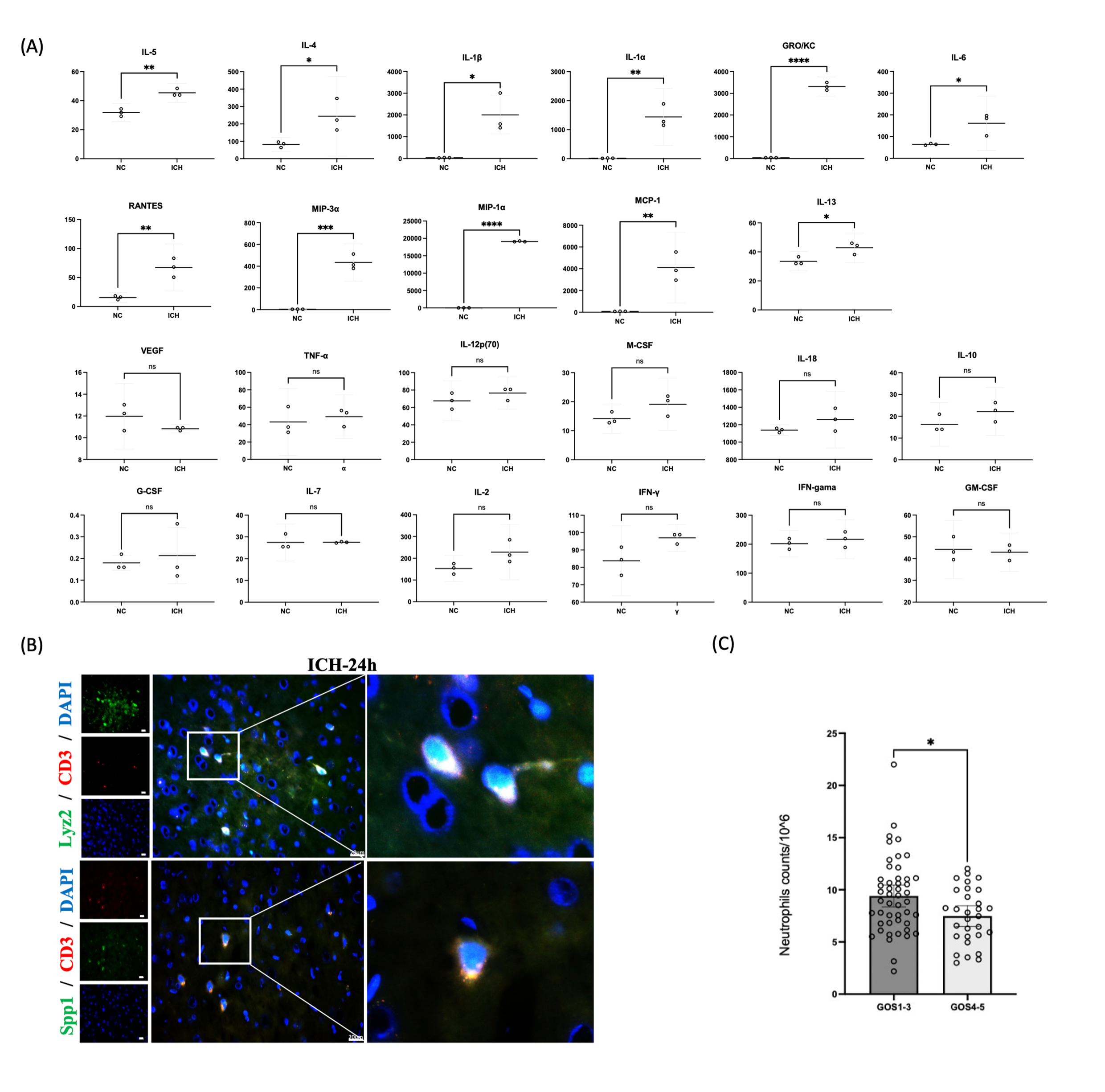


**Figure.S3. (A)** Inflammatory cytokine of rats brain tissue were assessed by Luminex®. (n=3 for each group. ns > 0.05,*P < 0.05, **P < 0.01, ***P < 0.001, ****P < 0.0001). **(B)** Double immunofluorescence staining showed CD3 and Lyz2 expressed on rat brain tissue at 24h after ICH, respectively. Scale bar =20 μm. (C) Neutrophil counts across patient groups with varying Glasgow Outcome Scale (GOS) scores. *P < 0.05.

**Table S1.** Canonical markers for each cell set.

**1.** Hao Y, Hao S, Andersen-Nissen E, et al. Integrated analysis of multimodal single-cell data. *Cell.* 2021/06/24/ 2021;184(13):3573-3587.e3529.

**2.** Stuart T, Butler A, Hoffman P, et al. Comprehensive Integration of Single-Cell Data. *Cell.* Jun 13 2019;177(7):1888-1902.e1821.

**3.** Butler A, Hoffman P, Smibert P, Papalexi E, Satija R. Integrating single-cell transcriptomic data across different conditions, technologies, and species. *Nat Biotechnol.* Jun 2018;36(5):411-420.

**4.** Satija R, Farrell JA, Gennert D, Schier AF, Regev A. Spatial reconstruction of single-cell gene expression data. *Nat Biotechnol.* May 2015;33(5):495-502.

**5.** Wu T, Hu E, Xu S, et al. clusterProfiler 4.0: A universal enrichment tool for interpreting omics data. *Innovation (Camb).* Aug 28 2021;2(3):100141.

**6.** Trapnell C, Cacchiarelli D, Grimsby J, et al. The dynamics and regulators of cell fate decisions are revealed by pseudotemporal ordering of single cells. *Nat Biotechnol.* Apr 2014;32(4):381-386.

**7.** Qiu X, Hill A, Packer J, Lin D, Ma YA, Trapnell C. Single-cell mRNA quantification and differential analysis with Census. *Nat Methods.* Mar 2017;14(3):309-315.

**8.** Qiu X, Mao Q, Tang Y, et al. Reversed graph embedding resolves complex single-cell trajectories. *Nat Methods.* Oct 2017;14(10):979-982.

**9.** Street K, Risso D, Fletcher RB, et al. Slingshot: cell lineage and pseudotime inference for single-cell transcriptomics. *BMC Genomics.* Jun 19 2018;19(1):477.

**10.** Gulati GS, Sikandar SS, Wesche DJ, et al. Single-cell transcriptional diversity is a hallmark of developmental potential. *Science.* Jan 24 2020;367(6476):405-411.

**11.** Wang Y, Wang R, Zhang S, et al. iTALK: an R Package to Characterize and Illustrate Intercellular Communication. *bioRxiv.* 2019:507871.

**12.** Jin S, Guerrero-Juarez CF, Zhang L, et al. Inference and analysis of cell-cell communication using CellChat. *Nat Commun.* Feb 17 2021;12(1):1088.

**13.** Ma Y, Zhou X. Spatially informed cell-type deconvolution for spatial transcriptomics. *Nat Biotechnol.* Sep 2022;40(9):1349-1359.

**14.** Moncada R, Barkley D, Wagner F, et al. Integrating microarray-based spatial transcriptomics and single-cell RNA-seq reveals tissue architecture in pancreatic ductal adenocarcinomas. *Nature Biotechnology.* 2020/03/01 2020;38(3):333-342.

**15.** Luo X, Li L, Zheng W, et al. HLY78 protects blood-brain barrier integrity through Wnt/beta-catenin signaling pathway following subarachnoid hemorrhage in rats. *Brain Res Bull.* Sep 2020;162:107-114.
